# Prophylactic prednisolone for the prevention of early and intermediate adverse effects of radioactive iodine therapy in patients with thyroid cancer: study protocol for a single-centre, phase II/III, randomized, double-blinded, placebo-controlled clinical trial

**DOI:** 10.1186/s13063-020-04744-x

**Published:** 2020-09-29

**Authors:** Umesh Jayarajah, Mahilal Wijekoon, Sanjeewa A. Seneviratne

**Affiliations:** 1 Department of Surgery, National Hospital of Sri Lanka, Colombo, Sri Lanka; 2 Department of Surgery, Faculty of Medicine, University of Colombo, P.O. Box 271, Kynsey Road, Colombo 8, Western Province Sri Lanka; 3 Apeksha Hospital, National Cancer Institute, Maharagama, Sri Lanka

**Keywords:** Prednisolone, Adverse effects, Radioactive iodine therapy, Thyroid cancer, Study protocol, Randomized control trial

## Abstract

**Background:**

Radioactive iodine (RAI) therapy is the standard adjuvant treatment for differentiated thyroid cancer (i.e. papillary and follicular). RAI is associated with troublesome early, intermediate and late adverse effects. Although glucocorticoids are used for the management of these adverse effects, there is little evidence regarding the effectiveness of prophylactic glucocorticoids to prevent these complications. This trial will evaluate the efficacy of a short course of prophylactic glucocorticoids in the prevention of adverse effects of RAI treatment in patients with differentiated thyroid cancer.

**Methods:**

A phase II/III, single-centre, randomized, double-blinded, placebo-controlled, parallel-arm clinical trial will be conducted. Patients with differentiated thyroid cancer who are referred to RAI therapy at the National Cancer Institute, Sri Lanka, will be randomized into two arms consisting of 200 patients each. The experimental group will receive prophylactic oral prednisolone 0.5 mg/kg and omeprazole 20 mg single dose 6 h before RAI therapy followed by oral prednisolone 0.5 mg/kg and omeprazole 20 mg daily for 3 days. The control group will receive oral placebo and omeprazole 20 mg single dose 6 h before RAI therapy followed by oral placebo and omeprazole 20 mg daily for 3 days. Clinically significant adverse effects assessed as related to RAI as well as prednisolone therapy and the quality of life parameters will be compared between the two groups.

**Discussion:**

If proven beneficial, this intervention can be incorporated into the standard practice to reduce early and intermediate adverse effects of RAI for thyroid cancer with a potential improvement of quality of life.

**Trial registration:**

Sri Lanka Clinical Trials Registry SLCTR/2020/009. Registered prospectively on 23 February 2020. Items of the WHO Trial Registration Data Set are provided in the supplementary file.

## Background

Radioactive iodine (RAI) ablation following thyroidectomy is the standard of care for most patients who are diagnosed with differentiated thyroid carcinoma [1–3]. RAI is administered with the primary objective of eliminating any residual thyroid cancer cells. The secondary objectives of RAI include improving the sensitivity of serum thyroglobulin assays during follow-up and improving the sensitivity of detection of both local and/or metastatic disease through functional and cross-sectional imaging [2, 4].

RAI is usually well-tolerated but is known to be associated with several adverse effects. Early adverse effects (up to 10 days after treatment) include local side effects (transient neck pain and swelling), salivary related (acute sialadenitis and xerostomia), nasal related (abnormalities in smell, epistaxis) and gastrointestinal side effects (ageusia, nausea, vomiting, stomatitis and ulcers). The incidence of early adverse effects is reported to be 10–20% for radiation thyroiditis and tumour swelling and 12–67% for acute radiation sialadenitis [5, 6]. Intermediate (10 days to 3 months) and late (after 3 months of treatment) adverse effects commonly include salivary-related side effects (recurrent chronic sialadenitis, xerostomia, salivary duct obstruction), eye-related adverse effects (xerophthalmia, epiphoria, conjunctivitis, nasolacrimal outflow obstruction) and dysfunction of body organs including pulmonary, gastrointestinal, haematopoietic systems and gonads [2, 4, 7, 8]. Chronic sialadenitis, xerostomia and abnormalities in taste or smell have been reported in approximately 20% and usually seen after multiple therapy. Other long-term adverse effects such as other organ involvement (pulmonary fibrosis, bone marrow suppression, etc.) are rare and seen in less than 1% [2, 6]. Furthermore, more serious late adverse effects such as second primary malignancies have also been reported, which however are rare. Although most of these complications are rarely serious or life threatening, they can have a significant impact on patients’ quality of life [4]. Radioactive iodine therapy is generally administered with high doses of a radioactive isotope of iodine (iodine-131), and in certain occasions, several cycles of treatment may be needed for an adequate response, particularly in cases of recurrent diseases or progressive iodine-avid metastatic diseases. Such instances substantially increase the possibility of adverse effects of RAI [4].

In a single-blind randomized study, Silberstein et al. assessed the efficacy of pilocarpine in the prevention of radiation sialadenitis and other adverse effects of RAI [9]. In this study, prophylactic glucocorticoids were given for a period of 3 days in both treatment and placebo arms. All patients received a combination of 8 mg of dexamethasone and 100 mg of dolasetron mesylate orally 2 h before therapy and every 12 hourly for another 5 doses after RAI ingestion in addition to the sialorrheic therapy with or without pilocarpine, 5 mg 8-hourly orally for 1 week [9]. Although pilocarpine was not associated with reduction in the adverse effects of RAI, the overall incidence of the adverse effects was considerably lower compared to previous data with only 5% acute and 1.66% chronic sialadenitis. This probably was due to the prophylactic glucocorticoids used in this study. However, no randomized study to date has studied the effects of steroids on the prevention of complications of RAI.

### Study rationale

Although glucocorticoids have been indicated in the management of certain adverse effects (early and intermediate), there is no clear evidence on its effectiveness in the prevention of adverse effects in the context of routine RAI treatment of thyroid cancer. Furthermore, due to the lack of robust evidence, the usage of glucocorticoids in the management varies depending on the individual perceptions and clinical experiences. Short-term glucocorticoid prophylaxis is inexpensive, is economically feasible and is well tolerated with minimal side effects. Thus, this study is aimed to assess the usefulness of glucocorticoids versus placebo for the prevention of early and intermediate adverse effects and the impact on quality of life following RAI treatment in thyroid cancer patients.

### Study objectives

#### Primary objectives

To estimate the impact of glucocorticoids on the incidence of predefined clinically significant early and intermediate adverse effects of RAI between patients treated with prophylactic prednisolone versus controls over a period of 3 months after completion of RAI.

#### Secondary objectives

1. To estimate the impact of glucocorticoids on the incidence of early and intermediate adverse effects of RAI during the first 3 months post therapy (all types, all grades, any duration).
2. To describe comparatively between treatment and control groups the onset, duration, recurrence and severity of early and intermediate adverse effects of RAI during the first 3 months after completion of RAI
3. To evaluate the impact of glucocorticoids on the incidence, severity and type of all adverse effects assessed as related to glucocorticoids
4. To evaluate the impact of glucocorticoids on the patient-reported outcomes related to adverse effects of RAI at 2 weeks and 3 months after completion of RAI
5. To study the impact on the quality of life at 2 weeks and 3 months, first overall between treatment groups, then focusing on patients who have experienced a complication of RAI

## Methods

### Study design

A phase II/III, single-centre, randomized, double-blinded, placebo-controlled, parallel-arm clinical trial with a superiority framework comparing prophylactic glucocorticoids versus a placebo will be performed with a minimum of 200 patients allocated to each arm.

### Study population

Adult patients with well-differentiated (i.e. papillary and follicular) thyroid cancer who are referred to RAI therapy at the National Cancer Institute, Sri Lanka.

### Subject eligibility

Patients will be included in the trial only if they meet all of the following inclusion criteria and none of the exclusion criteria. No exceptions, waivers or exemptions will be granted.

### Inclusion criteria

- Patients with histologically proven differentiated thyroid cancer following total thyroidectomy and are eligible for RAI therapy, referred to the National Cancer Institute, Sri Lanka, will be invited to participate in this study.
- No prior history of radiotherapy
- Age > 18 years
- An Eastern Cooperative Oncology Group (ECOG) performance status score of 0–1
- Patients must have normal marrow function as defined below:
  Leukocytes > 3000/μL
  Absolute neutrophil count > 1500/μL
  Platelets > 100,000/μL
- In females of the reproductive age group, exclusion of pregnancy before commencing RAI and avoiding future pregnancy for 6 months using contraceptives will be mandatory. Therefore, such patients who are capable of childbearing on adequate contraception will be included in this study.
- Available for follow-up and management in the study centre for at least 6 months
- Informed, voluntary, written consent
- Patients must sufficiently understand to fill in the quality of life and patient-reported outcome measures questionnaires.

### Exclusion criteria

- Those with absolute and relative contraindications for glucocorticoids such as uncontrolled diabetes mellitus, gastric and duodenal ulcers, immunosuppression, ongoing or active infections and chronic infective diseases
- History of previous RAI therapy
- Previous major head and neck surgery
- Patients with previous history of salivary gland diseases, such as sialadenitis, duct obstruction and calculi, and ophthalmological diseases such as xerophthalmia and conjunctivitis
- Uncontrolled concurrent illness including, but not limited to, symptomatic congestive heart failure, unstable angina pectoris, cardiac arrhythmia or psychiatric illness/social situations (within 12 months before the study) that would limit compliance with study requirements.
- Pregnant women and breast-feeding mothers are excluded as RAI therapy is contraindicated in such patients
- Diagnosed HIV-positive patients on combination antiretroviral therapy are ineligible as glucocorticoids may further suppress the immunity and increase the chances of opportunistic infection
- Prior diagnosis of cancer that was:
  More than 5 years prior to current diagnosis with subsequent evidence of disease recurrence or clinical expectation of recurrence is greater than 10%
  Within 5 years of current diagnosis with the exception of successfully treated basal cell or squamous cell skin carcinoma or carcinoma in situ of the cervix

### Recruitment strategies

All eligible patients referred to the National Cancer Institute, Sri Lanka, will be identified prospectively from the outpatient clinics during the first visit and will be invited to participate in the study. After obtaining informed written consent, they will be randomized to either intervention or placebo arm based on a computer-based random number generator. Randomization will be stratified by the total dose of radioactive iodine into 50–100, > 100 and < 200, and > 200 mCi. Recruitment will be allocated to each group in a ratio of 1:1:1. Patients will be recruited until a maximum number of 70 is achieved in each of the 3 arms. Patients who withdraw their consent to participate prior to response evaluation will be replaced.

### Study assessments

Participants will be given an information sheet and will be explained in detail about the study, and informed written consent will be taken prior to recruitment. The basic demographic information including age, sex, ethnicity and medical history including the details related to thyroid cancer (such as type, grade, stage, lymph node involvement), comorbidities and the current treatment will be collected using the clinic notes and interviewer-administered questionnaire. The contact details will also be collected for the purpose of follow-up. A general physical examination will be performed for a baseline assessment prior to administration of RAI. Basic haematological parameters including full blood count and renal functions will be performed before RAI treatment. A baseline quality of life scores which are EQ-5D-5L and FACT H&N will be measured [10]. Data collection tool is attached in the Supplementary file.

Patients will be stratified based on the dose of the RAI into three strata (50–100, > 100 and < 200, and > 200 mCi) based on the American Thyroid Association Guidelines 2015 [11]. From each group, patients will be randomized to two arms, the experimental and the placebo arms. All patients will be given a diary and asked to keep a log of any new symptoms after RAI therapy. Patients in both groups will be instructed to have sugar-free hard candy or gum in their mouths at all times when awake for a period of 1 week. Proton pump inhibitors are routine given in our setting to minimize dyspeptic symptoms which are reported to occur in around 50% and thus will be given to both arms [5]. This will also be helpful to minimize the gastrointestinal adverse effects of glucocorticoids. At least a fluid input of 2400 mL will be maintained during the first week after therapy. If a patient develops adverse effects of RAI, symptomatic management would be given, and the given treatment will be documented. Each patient will be reviewed at 2 weeks and 3 months by history, examination to assess for abovementioned side effects, for the measurement of quality of life scales and for possible complications of glucocorticoids. The onset, duration, recurrence and rate of each early and intermediate adverse effect will be assessed by a clinical interview and examination. For the grading of severity of the adverse effects, the Common Terminology Criteria for Adverse Effects version 4.0 will be used for an objective assessment by the clinician. Furthermore, the Patient-Reported Outcome version of the Common Terminology Criteria for Adverse Effects questionnaire will also be used to get the patient’s perspective regarding the presence of symptoms and their severity. After the study has been completed, patients will be subjected to standard routine follow-up for thyroid cancer.

### Study treatment and dosing schedule (dose, frequency and duration of the experimental treatment)

The treatment group includes oral prednisolone 0.5 mg/kg (maximum 40 mg) with omeprazole 20 mg single dose 6 h before RAI therapy and followed by daily (mane) with meals for 3 days. The control group includes oral placebo and omeprazole 20 mg single dose 6 h before RAI therapy and followed by daily (mane) with meals for 3 days. A placebo was chosen as the comparator as currently there is no alternative drug proven to reduce the adverse effects of RAI. If a dose is missed, the missed dose should be taken at the time of identification of the missed dose if it is during the same day and the next dose should be taken at the scheduled time.

The investigational drug formulation will be manufactured following all regulations. The placebo will be starch tablets of similar external features. The drug and the placebo will be specifically manufactured by the State Pharmaceutical Corporation of Sri Lanka for this study, and therefore, the quality of drug will be ensured.

The study investigators are responsible for enrolling participants, and this will be under the direct supervision of the principle investigator and the senior investigator. Blinding will be ensured by making sure that only one independent investigator will assign the drug and the placebo as either A or B randomly by computer-generated numbers and will be allocated to the next investigator as either drug A or drug B using sealed envelopes, who will be in charge of the drug administration. The details of identification of A and B will be concealed under lock and key until the data collection is complete to the other investigators. Thus, both the investigator and patient will be blinded. Unblinding will be permissible in the event of a severe adverse (CTCAE grade 4) event either due to RAI or prednisolone. In such instances, the investigators and clinicians taking care of the patients will decide on unblinding.

### Endpoint definitions

#### Primary outcome

Onset of one or more of predefined clinically significant adverse effects assessed as due to RAI during the 3-month period following completion of RAI treatment.

Side effects of RAI treatment will all be assessed clinically as there are no reliable tests to assess the side effects. The majority of the steroid-related side effects will also be assessed clinically, except capillary blood sugar level which will be assessed daily by the patient if on treatment for diabetes mellitus. Those without diabetes mellitus will not undergo routine capillary blood sugar monitoring as short-term, low-dose prednisolone does not warrant routine blood sugar monitoring in healthy individuals, in contrast to long-term administration [12]. The details and definitions of the adverse effects are given in Table 1. Clinically significant adverse effects are defined as the maximum severity of the adverse events assessed as related to RAI reaching the predefined level mentioned in Table 1 and/or if symptoms defined in Table 1 of any level of severity persisting for a prolonged (more than 2 weeks) duration.

**Table 1:** Definition of clinically significant outcomes

Related to	Classification	Symptom	Definition	CTCAE grade and definition
RAI	Local	Neck pain	A disorder characterized by marked discomfort sensation in the neck area	Grade 2 (impairment of instrumental activities of daily living) and above
RAI	Local	Neck swelling	A disorder characterized by marked enlargement in the neck area	Grade 2 (impairment of instrumental ADL) and above
RAI	Local	Dysphagia	A disorder characterized by difficulty in swallowing	Grade 2 (symptomatic and altered eating/swallowing) and above
RAI	Local	Dyspnoea	A disorder characterized by difficulty in breathing	Any grade
RAI	Oral	Sialadenitis	A disorder characterized by an inflammatory process involving the salivary gland	Grade 2 (moderate symptoms; oral intervention indicated) and above
RAI	Oral	Dry mouth	A disorder characterized by reduced salivary flow in the oral cavity	Grade 2 (moderate symptoms; oral intake alterations; unstimulated saliva 0.1 to 0.2 mL/min)
RAI	Oral	Oral mucositis	A disorder characterized by inflammation of the oral mucosal	Grade 2 (moderate pain; not interfering with oral intake; modified diet indicated) and above
RAI	Oral	Oral pain	A disorder characterized by a sensation of marked discomfort in the mouth, tongue or lip	Grade 2 (moderate pain; limiting instrumental ADL) and above
RAI	Oral	Dysgeusia	A disorder characterized by abnormal sensual experience with the taste of foodstuffs	Grade 2 (altered taste with change in diet (e.g. oral supplements), noxious or unpleasant taste, loss of taste) and above
RAI	Gastrointestinal	Nausea	A disorder characterized by a queasy sensation and/or the urge to vomit	Grade 2 (oral intake decreased without significant weight loss, dehydration or malnutrition) and above
RAI	Gastrointestinal	Vomiting	A disorder characterized by the reflexive act of ejecting the contents of the stomach through the mouth	Grade 2 (3–5 episodes (separated by 5 min) in 24 h) and above
RAI/glucocorticoids	Gastrointestinal	Dyspepsia	A disorder characterized by an uncomfortable, often painful feeling in the stomach, resulting from impaired digestion. Symptoms include burning stomach, bloating, heartburn, nausea and vomiting	Grade 2 (moderate symptoms; medical intervention indicated) or above
RAI/glucocorticoids	Gastrointestinal	Abdominal pain	A disorder characterized by a sensation of marked discomfort in the abdominal region	Grade 2 (moderate pain; limiting instrumental ADL)
RAI	Eye	Xerophthalmia	A disorder characterized by dryness of the cornea and conjunctiva	Grade 2 (symptomatic; multiple agents indicated; limiting instrumental ADL) and above
RAI	Eye	Epiphoria	A disorder of excessive tearing in the eyes	Grade 2 (intervention indicated) and above
RAI	Eye	Conjunctivitis	A disorder characterized by inflammation, to the conjunctiva of the eye	Grade 2 (symptomatic; topical intervention indicated (e.g. antibiotics); limiting instrumental ADL) and above
Glucocorticoids	General	Infections within 2 weeks	A disorder characterized by an infectious process	Grade 2 (any infection that needs intervention) or above
Glucocorticoids	General	Hyperglycaemia during the first 3 days	A disorder characterized by laboratory test results that indicate an elevation in the concentration of blood sugar	Grade 3 (> 250–500 mg/dL; > 13.9–27.8 mmol/L; hospitalization indicated) or above
Glucocorticoids	Central nervous system	Altered sensorium	A disorder characterized by functional disturbances of sensory neurons resulting in abnormal cutaneous sensations of tingling, numbness, pressure, cold and warmth that are experienced in the absence of a stimulus	Grade 2 (moderate symptoms limiting instrumental ADL) and above
Glucocorticoids	Central nervous system	Sleep disturbances	A disorder characterized by excessive or reduced sleepiness or difference in sleep pattern	Grade 2 (moderate disturbance) or above
Glucocorticoids	Central nervous system	Mood changes	A disorder characterized by a state of restlessness, irritability or sadness	Grade 3 (moderate symptoms; hospitalization not indicated) or above

*ADL* activities of daily living, *CTCAE* Common Terminology Criteria for Adverse Effects version 4.0

#### Secondary endpoints

1. Onset of any type, grade and duration of early and intermediate adverse effects assessed as due to RAI
2. Onset, duration, recurrence and severity (CTCAE) of early and intermediate adverse effects of RAI defined in Table 1
3. Onset, incidence, severity, duration and type of adverse events assessed in relation to glucocorticoids
4. Patient-reported outcomes related to adverse effects of RAI and glucocorticoids: NCI PRO-CTCAE at 2 weeks and 3 months after completion of RAI
5. Quality of life scores: EQ-5D-5L and FACT H&N

(CTCAE Common Terminology Criteria for Adverse Effects, PRO-CTCAE Patient-Reported Outcomes-Common Terminology Criteria for Adverse Effects version 4.0, FACT H&N Functional Assessment of Cancer Therapy Head and Neck)

### Assessment of response

Assessment of responses will be done by validated instruments in the native language including EQ-5D-5L and translated and validated questionnaires. These include FACT H&N and PRO-CTCAE. Quality of life surveys will be carried out using EQ-5D-5L which is a simple, non-specific, generic health-related quality of life instrument that is self-administered and is widely used as a patient-reported outcome measure. It consists of five health dimensions (mobility, self-care, usual activities, pain/discomfort and anxiety/depression). This instrument has been validated in Sri Lankan participants and was found to have satisfactory psychometric properties [13].

FACT Head and Neck (FACT H&N) is a simple, practical, clinician-rated assessment tool consisting subscales related to head and neck malignancy. FACT H&N scale concerns include oral comfort, breathing, voice, eating, appearance, pain and communication. Results indicate that the scale is reliable across the subscales and sensitive to functional differences across a spectrum of head and neck cancers with [14].

The National Cancer Institute’s Patient-Reported Outcomes version of the Common Terminology Criteria for Adverse Events (PRO-CTCAE) is a promising tool to provide a standard yet flexible method to assess symptomatic adverse events in patients with cancer. Thus, this is a patient-reported version of the CTCAE clinical grading tool which would be used to get the patient’s perspective regarding the adverse effects of interest. The PRO-CTCAE is publicly available for free use for clinical research [15].

### Screening evaluation

- Those who fulfil the eligibility criteria will be invited to participate in the study.
- Clinical examination will include that of oral examination and eye examination to look for evidence of prior diseases similar to the adverse effects mentioned in Table 1.
- Full blood count will be done in all patients to confirm eligibility.
- Studies will be completed within 4 weeks prior to enrolling onto the protocol.

### Registration procedures

- All patients will be registered on study once study consent has been obtained and eligibility has been confirmed by the investigators. Registration and randomization will be performed 2 weeks prior to the planned date of RAI.

#### Randomization (or stratification) procedures

- At study entry, patients will be randomly allocated to one of the two arms, the intervention arm and the placebo arm. Randomization will be balanced (1:1), using permuted blocks, and stratified according to the total dose of RAI given for stratification into three groups. Criteria for stratification will be 50–100, > 100 and < 200, and > 200 mCi.
- An independent investigator will be responsible for randomization of treatment using an online randomization software, ensuring concealment of treatment allocation for the next patient.

#### Baseline evaluation

- Clinical assessment: Symptom analysis and oral and eye examination
- EQ-5D-5L and FACT H&N
- These will be completed within 2 weeks before the first dose of drug or registration.

#### Drug administration

The treatment group will receive oral prednisolone 0.5 mg/kg and omeprazole 20 mg single dose 6 h before RAI therapy and followed by prednisolone 0.5 mg/kg and omeprazole 20 mg daily for 3 days. The control group will receive oral placebo and omeprazole 20 mg single dose 6 h before RAI therapy and followed by placebo and omeprazole 20 mg daily for 3 days.

### Follow-up procedures

All patients will be given a symptom diary and instructions will be given how to maintain it. Patients will be instructed to bring the diary at each follow-up visit through telephone reminders. If they develop a problematic adverse effect, they will be instructed to contact the investigator. During visit 1 at 2 weeks, clinical interviews will be conducted to assess the adverse effects including oral and eye examination. The adverse effects will be recorded based on the CTCAE grading system by the investigator and also by patient-reported outcome measures NCI PRO-CTCAE. The patients will be reminded about the follow-up visits by phone calls. Data on patients who discontinue the trial or deviate from the protocol will be recorded.

The quality of life instruments including EQ-5D-5L and FACT H&N will be completed by the patient. During visit 2 at 3 months, the same assessment will be conducted with more emphasis on the intermediate adverse effects.

Criteria for removal from protocol therapy include participant requests to be withdrawn from therapy and unacceptable toxicity such as infections, hyperglycaemia or severe dyspepsia. Furthermore, a patient who would prematurely stop the treatment will not be withdrawn from the study unless he/she requests to be withdrawn from the study. There are no specific concomitant treatments or interventions that are permitted or prohibited during the trial.

Off-study criteria include completed 3-month follow-up period, subject withdrawal from the follow-up period and death.

### Feasibility

In Sri Lanka, thyroid cancer has become the third most common cancer next to breast and oral cancer [16–18]. Furthermore, the incidence is increasing at a considerable rate. Each year, around 1600 patients are diagnosed of thyroid cancer of which around 500 are referred to the National Cancer Institute for RAI [18].

### Protocol waiver/deviations/violation

Any deviations from the protocol will be described and justified as a protocol amendment or in the final report. Changes and amendments to the protocol will only be made by the study committee. Approval of amendments by the institutional ethical review committee will be required prior to their implementation. The investigators will obtain approval/advice for the revised consent form from the ethics committee prior to implementation of the change/s. In addition, changes to the questionnaires and re-consenting, if required, will be incorporated in the amendment. Any changes to the protocol will be communicated to interested parties such as investigators and the trial registry through the official email address and also directly during research team meetings either in person or online. The investigator will not implement any changes to or deviations from the protocol except where it is essential to eliminate immediate hazard(s) to study subject(s).

The privacy and confidentiality of medical information will be maximized. There will be no biological specimens collected for this study. The study will be conducted in accordance with applicable privacy acts and regulations. No participant identifiers will be used in data collection. However, age, ethnicity, sex, diagnosis and stage will be collected. All data generated in this study will remain confidential and only anonymized data will be used in publications. Participants will be informed that completed questionnaires will only be seen by authorized members of the research team. Study data will be collected on the questionnaire forms and will then be transferred into a password-protected computerized database maintained at the Department of Surgery, Faculty of Medicine, Colombo. Data will be kept for a period of 1–3 years after completion of the study and thereafter deleted. Only data collectors and study investigators will have access to the database. Trial results will be communicated to the participants and via scientific publications in peer-reviewed journals and presentations at scientific fora.

### Safety of caregivers and health care providers

Patients treated with high doses of radioactive iodine are isolated for 3–5 days to prevent exposure to caregivers (including health care staff). To prevent exposure of caregivers and health care providers to radiation, the tablets for the 3-day period will be given to the patient before administration of radioactive iodine and will be instructed to take the medications at the scheduled time. Patients will be monitored whether they are taking the medications through phone calls. This will minimize the exposure of care givers and health care staff.

All adverse events, which occur whilst the participant is enrolled on the trial, will be reported in the patients’ medical records and recorded on the relevant case form. The Common Terminology Criteria for Adverse Events (CTMAE version 4.0—see appendices) will be used to grade the severity of an event.

### Data and Safety Monitoring Board (DSMB)

The primary responsibilities of the DSMB will include periodical review and evaluation of the study data for participant safety, study conduct and progress and make recommendations to investigators and relevant other parties concerning the continuation, modification or termination of the trial. The DSMB will consider study-specific data as well as relevant background data about the differentiated thyroid CA, RAI treatment, use of prednisolone and patient population under study.

During the trial, the DSMB will review cumulative study data to evaluate safety of prednisolone, study conduct and scientific validity and integrity of the trial. DSMB members will look into the timeliness, completeness and accuracy of the data to ensure safety and welfare of study participants. The DSMB will also assess the performance of overall study operations and any other relevant issues, as necessary. Members of the DSMB will meet every 8 weeks to review the study data and make recommendation to the research team on conduct of the study. The DSMB will include three independent experts in the field (Supplementary file).

### Data analysis

#### Sample size calculation

The reported rates of radiation thyroiditis are approximately 20% whilst acute radiation sialadenitis is 12–67% and chronic sialadenitis and xerostomia were 20% [5, 6]. Based on these available data, we assume that clinically significant adverse effects will be reported at 3 months in 20% of patients. At a 2-sided 5% alpha level, a total of 200 patients in each group is required to ensure an 80% power if glucocorticoids are associated with a 10% absolute reduction (10% vs. 20%).

### Statistical analysis

Analyses will be performed in accordance with the intention-to-treat analysis principle.

All randomized participants randomized to study and control groups will be included in the main analyses irrespective of protocol adherence (i.e. intention-to-treat analysis). An additional per-protocol analysis will also be conducted only including the patients who adhered to the treatment protocol with completed follow-up. For each patient, the maximum grade for all types of events will be calculated over the whole observation period. The maximum grades per patient will be tabulated, first considering all adverse effects, then focusing on clinically relevant adverse effects as defined in Table 1 (primary endpoint). The proportion of patients who have experienced clinically relevant adverse effects will be estimated in each group. Difference of proportions between the 2 treatment groups as well as odds ratio will be provided to estimate the impact of glucocorticoids on the risk of clinically significant RAI therapy-related adverse effects. All these estimates will be provided with their 95% confidence intervals. Analyses will be adjusted on the stratification factors. Comparisons between groups on binary endpoints will be performed using a logistic regression, and comparisons between groups on continuous endpoints will be performed using *t* tests. The *p* value will be evaluated at the 5% level of significance. No adjustment will be made for multiple comparisons associated with secondary analyses, but these will be evaluated in proper context and fully reported.

## Discussion

The adverse effects of RAI may be long lasting and may considerably impact the quality of life of patients. The treatment of adverse effects of RAI is mainly symptomatic. This includes proper hydration, use of sialagogues, antiemetics and proton pump inhibitors, and in cases of inflammatory tumour expansion, glucocorticoids may be administered [5, 6]. This is the first randomized control clinical trial designed to assess the effectiveness of prophylactic steroids in reducing the adverse effects of RAI. We hypothesize that a short course of prophylactic prednisolone would be beneficial in reducing the early and intermediate adverse effects of RAI.

Glucocorticoids have significant anti-inflammatory and immunosuppressive properties through its genomic and non-genomic effects [19]. Due to its effect of reducing serum IgG after RAI therapy, this might reflect a decrease of any potential immune-mediated cytotoxicity elicited by the radiation [20]. Furthermore, glucocorticoids may also have a direct radioprotective effect by reducing the intracellular oxidative stress, but it needs further clarification [20]. Also, inflammatory tumour expansion is implicated in the pathogenesis of certain adverse effects such as neck swelling and tracheal compression [6]. Therefore, a short course of glucocorticoids is recommended in patients with symptoms of neck swelling and discomfort following RAI which is seen in large thyroid remnants [6, 21]. Furthermore, glucocorticoid prophylaxis has been recommended in cases where thyroid tumour deposits are suspected near vital structures or in confined anatomical spaces such as the brain, spinal cord, lungs or bone to prevent or minimize the effect of inflammatory tumour expansion [6, 21]. Probable adverse effects of glucocorticoids, such as poorly controlled diabetes mellitus, gastric and duodenal ulcers or electrolyte disorders, should be considered when using glucocorticoids [6]. However, such adverse effects are rare when used for a short duration following exclusion of patients with contraindications for glucocorticoids.

Benefits to subjects will be directly related to the reduction of adverse effects of RAI provided that glucocorticoids are efficacious in reducing such effects. If the glucocorticoids are proven effective, this prophylactic measure could be incorporated to routine practice to minimize adverse effects of RAI. Potential risks would include potential adverse effects of short course of glucocorticoids; however, proton pump inhibitors will be concurrently administered to minimize gastrointestinal adverse effects. Other rare adverse effects include transient increased susceptibility to infections, transient hyperglycaemia, sleep disturbances, altered sensorium and mood changes. In the event of adverse effects to the subjects, medical or professional intervention will be sought. All participants will be educated about the adverse effects of RAI and the safety precautions that should be taken after administration.

If proven to be effective, short-term steroid prophylaxis could be made standard with the administration of RAI treatment for differentiated thyroid cancer which would minimize side effects and improve quality of life.

### Trial status

Recruitment for this trial (version 2.0 on 21 November 2019) has not been commenced yet. The anticipated date for commencement is on 1 November 2020.

## Supplementary information

**Additional file 1.** Concept sheet: Summary of the main components of the methodology.

**Additional file 2.** Flow chart of study protocol.

**Additional file 3.** WHO trial registry data set, DSMB members and data collection form.

## Data Availability

Not applicable, no datasets are included in this study protocol.

## Abbreviations

RAI: Radioactive iodine
ECOG: Eastern Cooperative Oncology Group
HIV: Human immunodeficiency virus
FACT H&N: Functional Assessment of Cancer Therapy Head and Neck
CTCAE: Common Terminology Criteria for Adverse Effects
PRO-CTCAE: Patient-Reported Outcomes-Common Terminology Criteria for Adverse Effects
DSMB: Data and Safety Monitoring Board
