# Supplementary material for: Prophylactic prednisolone for the prevention of early and intermediate adverse effects of radioactive iodine therapy in patients with thyroid cancer: study protocol for a single-centre, phase II/III, randomized, double-blinded, placebo-controlled clinical trial
Source: Trials. 2020 Sep 29;21:812. doi: 10.1186/s13063-020-04744-x (PMC7526358; doi:10.1186/s13063-020-04744-x)
Supplement: Supplementary file 1 — Additional file 1. Concept sheet: Summary of the main components of the methodology. [file 13063_2020_4744_MOESM1_ESM.docx]

# Concept Sheet

| **Title** | Prophylactic prednisolone for the prevention of early and intermediate adverse effects of radioactive iodine therapy in patients with thyroid cancer |
| --- | --- |
|  |  |
| **Background and rationale** | Radioactive iodine (RAI) therapy is the standard adjuvant treatment for well-differentiated thyroid cancer. RAI is associated with troublesome adverse effects, which include early, intermediate and late adverse effects. Although glucocorticoids are recommended for the management of these adverse effects, there is little evidence regarding the effectiveness of prophylactic glucocorticoids to prevent these complications. |
|  |  |
| **Aim** | To evaluate the efficacy of prophylactic short course of glucocorticoids in the prevention of adverse effects of RAI treatment in patients with thyroid cancer. |
|  |  |
| **Objectives (endpoints)**  **Primary**      **Secondary** | 1. To estimate the impact of glucocorticoids on the incidence of predefined clinically significant early/intermediate adverse effects of RAI between patients treated with prophylactic prednisolone versus placebo over a period of 3 months after completion of RAI  1. To estimate the impact of glucocorticoids on the proportion of patients developing early and intermediate adverse effects due to RAI in the first 3 months after completion of RAI (all types, all grades, any duration)  2. To describe comparatively between treatment and control groups, the onset, recurrence, duration and severity of early and intermediate adverse effects of RAI in the first 3 months after completion of RAI  3. To estimate the impact of glucocorticoids in terms of the incidence, severity, duration and type of adverse events assessed in relation to glucocorticoids  4. To evaluate the impact of glucocorticoids on the patient reported outcomes related to adverse effects of RAI and/or glucocorticoids at 2 weeks and 3 months after completion of RAI  5. To evaluate the impact on the quality of life at 2 weeks and 3 months, first, overall between treatment groups, then focusing on patients who have experienced a complication of RAI |
|  |  |
| **Hypothesis** | Glucocorticoids are associated with a reduction of occurrence of adverse effects of radioactive iodine. |
| **Population and setting** | Patients with papillary and follicular thyroid cancer who are referred to RAI therapy at the National Institute of Cancer, Sri Lanka |
|  |  |
| **Interventions** | Experimental: Prophylactic oral (prednisolone 0.5mg/kg and omeprazole 20mg) single dose 6 hours before RAI therapy and followed by oral (prednisolone 0.5mg/kg and omeprazole 20mg) daily for 3 days  Control: Oral (Placebo + omeprazole 20mg) single dose 6 hours before RAI therapy and followed by oral (Placebo + omeprazole 20mg) daily for 3 days |
|  |  |
| **Study design** | A phase II/III, single centre, randomized, double blinded, placebo controlled, parallel arm clinical trial |
|  |  |
| **Outcomes and measures** | 1. Clinically significant adverse effects assessed as related to RAI as defined in Table 1 of the protocol 2. Early and intermediate adverse effects due to RAI 3. Onset, recurrence, duration and severity (CTCAE) of early and intermediate adverse effects of RAI 4. Onset, incidence, severity, duration and type of adverse events assessed in relation to glucocorticoids 5. Patient reported outcomes related to adverse effects of RAI and glucocorticoids: NCI- PRO-CTCAE at 2 weeks and 3 months after completion of RAI 6. Quality of life scores: [EQ-5D-5L](https://euroqol.org/wp-content/uploads/2016/09/EQ-5D-5L_UserGuide_2015.pdf) and FACT H&N |
|  |  |
| **Study procedures** | Treatments are randomized between two arms, the prophylactic prednisolone group and the placebo group, which will be done within 2 weeks prior to RAI therapy. Randomisation will be stratified by the dose of radioactive iodine 50-100, >100 and <200, and >200 milliCuries. Other measures to avoid adverse effects such as sialagogues will be given to both groups. If patient develops adverse effects of RAI, symptomatic management would be given and the given treatment will be documented. Follow-up visits for safety evaluation are planned at 2 weeks and 3 months after the end of RAI therapy. Patients will be instructed to maintain a diary to report adverse effects and will be instructed to contact the investigators in case of any troublesome adverse effects develop. |
|  |  |
| **Statistical considerations** | Based on previous experience, we assume that clinically significant adverse effects will be reported at 3 months in 20% of patients. Using a chi^2^ test to compare both treatment groups at a 2-sided 5% alpha level, a total of 200 patients in each group is required to ensure an 80% power if glucocorticoids are associated with a 10% absolute reduction (10% vs. 20%). |
|  |  |
| **Feasibility** | 1.Approximately 600-700 patients are referred to National Cancer Institute for RAI treatment each year  2. Glucocorticoids are readily available at low cost and economically feasible. |
|  |  |
| **Significance** | If proven beneficial, this can be incorporated into the standard practice to prevent the early and intermediate adverse effects of RAI for thyroid cancer with improvement of quality of life. |
|  |  |
| **Funding** | Proposal will be submitted to the University Grants Commission for possible funding for this study. |
|  |  |
| **Risks** | Patients will be exposed to adverse effects of glucocorticoids. However, they are minimal if used as a short course |
