# Supplementary material for: Prophylactic prednisolone for the prevention of early and intermediate adverse effects of radioactive iodine therapy in patients with thyroid cancer: study protocol for a single-centre, phase II/III, randomized, double-blinded, placebo-controlled clinical trial
Source: Trials. 2020 Sep 29;21:812. doi: 10.1186/s13063-020-04744-x (PMC7526358; doi:10.1186/s13063-020-04744-x)
Supplement: Supplementary file 2 — Additional file 2. Flow chart of study protocol. [file 13063_2020_4744_MOESM2_ESM.docx]

# Study schema- A single centre, phase II/III, randomised, double blind, placebo controlled clinical trial

First follow up – At 14 days after RAI

Second follow up– at three months after RAI

**Allocation**

Exclusion criteria

- Compelling indication for steroid prophylaxis
- History of RAI therapy
- Contraindications for glucocorticoids
- Previous other major head and neck surgery or severe comorbid illnesses affecting quality of life
- History of salivary gland diseases or xerophthalmia, chronic conjunctivitis

**Enrolment**

Eligibility – Adults patients with histologically proven well-differentiated papillary and follicular thyroid cancer following total thyroidectomy and are eligible for RAI therapy referred to the National Institute of Cancer, Sri Lanka.

Stratified randomization based on the dose of RAI 50-100, >100 and <200, and >200 milliCuries

Prophylactic oral (prednisolone 0.5mg/kg (max:40mg) and omeprazole 20mg) single dose 6 hours before RAI therapy and followed by (prednisolone 0.5mg/kg (max:40mg) and omeprazole 20mg) daily for 3 days.

Oral (Placebo + Omeprazole 20mg) single dose 6 hours before RAI therapy and followed by (Placebo + Omeprazole 20mg) daily for 3 days.

**Follow-Up**

First follow up – At 14 days after RAI

Second follow up – at three months after RAI

**Primary endpoints**

Proportion of patients developing predefined clinically significant adverse effects due to RAI will be compared in both arms.
