# Supplementary material for: Prophylactic prednisolone for the prevention of early and intermediate adverse effects of radioactive iodine therapy in patients with thyroid cancer: study protocol for a single-centre, phase II/III, randomized, double-blinded, placebo-controlled clinical trial
Source: Trials. 2020 Sep 29;21:812. doi: 10.1186/s13063-020-04744-x (PMC7526358; doi:10.1186/s13063-020-04744-x)
Supplement: Supplementary file 3 — Additional file 3. WHO trial registry data set, DSMB members and data collection form. [file 13063_2020_4744_MOESM3_ESM.docx]

**Prophylactic prednisolone for the prevention of early and intermediate adverse effects of radioactive iodine therapy in patients with thyroid cancer: a single centre, phase II/III, randomized, double blinded, placebo controlled clinical trial**

**Supplementary material**

**WHO Trial Registration Data Set**

Primary Registry and Trial Identifying Number

Sri Lanka Clinical Trials Registry: SLCTR/2020/009

Date of Registration in Primary Registry

23 Feb 2020

Scientific Title of Trial

Prophylactic prednisolone for the prevention of early and intermediate adverse effects of radioactive iodine therapy in patients with thyroid cancer: a single centre, phase II/III, randomized, double blinded, placebo controlled clinical trial

Public Title of Trial

Prophylactic prednisolone for the prevention of early and intermediate adverse effects of radioactive iodine therapy in patients with thyroid cancer: a single centre, phase II/III, randomized, double blinded, placebo controlled clinical trial.

Disease or Health Condition(s) Studied

Differentiated Thyroid cancer, Preventive interventions for side effects of radioactive iodine

Secondary Identifying Numbers

Universal Trial Number: U1111-1244-9739

**Trial Details**

What is the research question being addressed?

Does a short course of prophylactic prednisolone prevent short-term and medium term side effects of radioactive iodine in patients with differentiated thyroid cancer?

Type of study

Interventional

Study design

Allocation

Randomized controlled trial

Masking

Double blinded: Participants, Investigators

Control

Placebo

Assignment

Parallel

Purpose

Prevention

Study Phase

Phase 2-3

Intervention(s) planned

Study Setting: Apeksha Hospital (National Institute of Cancer), Maharagam, Sri Lanka.

After obtaining informed written consent from patients who accept the invitation, they will be randomized to either intervention arm or placebo arm based on a computer based random number generator. Randomization will be stratified by the total dose of radioactive iodine 50-100, >100 and <200, and >200 milliCuries. Recruitment will be allocated to each group in a ratio of 1:1:1. Patients who withdraw their consent to participate prior to response evaluation will be replaced. Participants will be given an information sheet and will be explained in detail about the study, and informed written consent will be taken prior to recruitment. The basic demographic information such as age, sex, ethnicity and medical history including the details related to thyroid cancer (such as type, grade, stage, lymph node involvement), comorbidities and the current treatment will be collected using the clinic notes and interviewer administered questionnaire. The contact details will also be collected for the purpose of follow up. A general physical examination will be performed for a baseline assessment prior to administration of radioactive iodine (RAI). Basic haematological parameters including full blood count and renal functions will be performed before RAI treatment. A baseline quality of life scores which include Euro-Qol (EQ-5D-5L)and Functional Assessment of Cancer Therapy-Head and Neck (FACT H&N) will be measured. Patients will be stratified based on the dose of the RAI into three strata (50-100, >100 and <200, and >200 milliCuries) based on the American Thyroid Association Guidelines 2015. Then from each group, patients will be randomised to two arms, the experimental arm and the placebo arm.

Experimental arm: Prophylactic oral (prednisolone 0.5mg/kg and omeprazole 20mg) single dose 6 hours before RAI therapy and followed by oral (prednisolone 0.5mg/kg and omeprazole 20mg) daily for 3 days

Control/ placebo arm: Oral (Placebo + omeprazole 20mg) single dose 6 hours before RAI therapy and followed by oral (Placebo + omeprazole 20mg) daily for 3 days. Starch tablets of similar shape, size and colour would be used as placebo.

All patients will be given a diary and asked to keep a log of any new symptoms after RAI therapy. Patients in both groups will be instructed to have sugar-free hard candy or gum in their mouths at all times when awake for a period of one week. Proton pump inhibitors are routinely given in our setting to minimise dyspeptic symptoms which is reported to occur in around 50% and thus will be given to both arms. This will also be helpful to minimise the gastrointestinal adverse effects of glucocorticoids. At least a fluid input of 2,400 mL will be maintained during the first week after therapy. If patient develops adverse effects of RAI, symptomatic management would be given and the given treatment will be documented. After completion of the study patients will subjected to standard routine follow up for thyroid cancer.

Inclusion criteria

a) Patients with histologically proven differentiated papillary and follicular thyroid cancer following total thyroidectomy and are eligible for RAI therapy, referred to the National Institute of Cancer, Sri Lanka will be invited to participate in this study. b) Without any history of radiotherapy c) Age >18 years d) An ECOG performance status score of 0-1 e) Patients must have normal marrow function as defined below: leukocytes >3,000/mcL absolute neutrophil count >1,500/mcL platelets >100,000/mcL f) In females of the reproductive age group, exclusion of pregnancy before commencing RAI and avoiding future pregnancy for 6 months using contraceptives is mandatory. Therefore, such patients who are capable of child bearing on adequate contraception will be included in this study. g) Available for follow up and management in the study centre for at least 6 months h) Informed, voluntary, written consent i) Patients must sufficiently understand English, Sinhala or Tamil to fill in the quality of life and patient reported outcome measures.

Exclusion criteria

a). Those with absolute and relative contraindications for glucocorticoids such as uncontrolled diabetes mellitus, gastric and duodenal ulcers, immunosuppression, ongoing or active infections, chronic infective diseases b). History of previous RAI therapy c). Previous major head and neck surgery d). Patients with severe debilitating diseases that can affect the quality of life. e). Patients with previous history of salivary gland diseases such as sialadenitis, duct obstruction, calculi, and ophthalmological diseases such as xerophthalmia and conjunctivitis f) Uncontrolled concurrent illness including, but not limited to, symptomatic congestive heart failure, unstable angina pectoris, cardiac arrhythmia, or psychiatric illness/social situations (within 12 months before study) that would limit compliance with study requirements. g) Pregnant women and breast feeding mothers are excluded as RAI therapy is contraindicated in such patients. h) Diagnosed HIV-positive patients on combination antiretroviral therapy are ineligible as glucocorticoids may further suppress the immunity and increase the chances of opportunistic infection i) Prior diagnosis of cancer that was: • More than 5 years prior to current diagnosis with subsequent evidence of disease recurrence or clinical expectation of recurrence is greater than 10% • Within 5 years of current diagnosis with the exception of successfully treated basal cell or squamous cell skin carcinoma or carcinoma in situ of the cervix

Primary outcome(s)

| To estimate the impact of glucocorticoids on the incidence of predefined clinically significant early/intermediate adverse effects of RAI (according to the Common Terminology Criteria for Adverse Events v4.0)between patients treated with prophylactic prednisolone versus placebo | *[2 weeks and 3 months after completion of RAI ]* |
| --- | --- |

Key Secondary outcome(s)

| 1. To estimate the impact of glucocorticoids on the proportion of patients developing early and intermediate adverse effects due to RAI in the first 3 months after completion of RAI (all types, all grades, any duration) | *[3 months ]* |
| --- | --- |
| 2. To describe comparatively between treatment and control groups, the onset, recurrence, duration and severity of early and intermediate adverse effects of RAI in the first 3 months after completion of RAI | *[3 months ]* |
| 3. To estimate the impact of glucocorticoids in terms of the incidence, severity, duration and type of adverse events assessed in relation to glucocorticoids | *[3 months ]* |
| 4. To evaluate the impact of glucocorticoids on the patient reported outcomes related to adverse effects of RAI and/or glucocorticoids at 2 weeks and 3 months after completion of RAI | *[2 weeks and 3 months ]* |
| 5. To evaluate the impact on the quality of life (Euro-Qol (EQ-5D-5L) and Functional Assessment of Cancer Therapy-Head and Neck (FACT H&N)), first, overall between treatment groups, then focusing on patients who have experienced a complication of RAI. | *[2 weeks and 3 months ]* |

Target number/sample size

398 (199 in both intervention and placebo arms)

Countries of recruitment

Sri Lanka

Date of First Enrollment

2021-02-01

Sources of Monetary or Material Support

Pending

Primary Sponsor

Pending

Secondary Sponsor(s)

Pending

Recruitment status

Pending

Regulatory approvals

National Medicine Regulatory Authority approved

**Ethics Review**

Status

Approved

Date of Approval

2019-11-21

Approval number

EC-19-076

Details of Ethics Review Committee

| Name: | Ethics Review Committee of Faculty of Medicine, University of Colombo, Sri Lanka |
| --- | --- |
| Institutional Address: | PO box 271, Kynsey Road ,Colombo 8, Sri Lanka |
| Telephone: | 94112695300 ext 240 |
| Email: | ethicscommitteemfc@gmail.com |

**Contact & Sponsor Information**

Contact person for Scientific Queries/Principal Investigator

Umesh Jayarajah
Registrar in Surgery
National Hospital of Sri Lanka, Colombo 10
+94112691111
+94766808625

[umeshe.jaya@gmail.com](mailto:umeshe.jaya@gmail.com)

Contact Person for Public Queries

Sanjeewa A Seneviratne
Senior Lecturer in Surgery and Honorary Consultant Surgeon
Department of Surgery, Faculty of Medicine, University of Colombo. PO Box 271, Kynsey Road, Colombo 08, Sri Lanka
+94 112 671846
+94 772 645377

[sanjeewa@srg.cmb.ac.lk](mailto:sanjeewa@srg.cmb.ac.lk)

Primary study sponsor/organization

Faculty of Medicine, University of Colombo
Department of Surgery
Department of Surgery, Faculty of Medicine, University of Colombo. PO Box 271, Kynsey Road, Colombo 08, Sri Lanka
+94 112 671846
+94 112 671846

<https://med.cmb.ac.lk/surgery/>

Secondary study sponsor (If any)

IPD sharing statement

All individual participant data collected during the trial, after de-identification will be shared upon reasonable request. The study protocol and the statistical analysis plan will be shared. Data will be shared following publication of the results. Data will be shared among investigators whose proposed use of the data has been approved by an independent review committee identified for this purpose. Data will be shared to achieve the aims in an approved proposal and for individual participant data meta-analysis. Proposals should be directed to sanjeewa@srg.cmb.ac.lk . To gain access, data requestors will need to sign a data access agreement. Data will be available for 5 years from the time of publication at the Department if Surgery data warehouse. Information regarding submitting proposals and accessing data may be found by e-mailing the supervisor at sanjeewa@srg.cmb.ac.lk

Date of study completion

Pending

Summary results

Pending

**Data & Safety Monitoring Board (DSMB)**

The DSMB will include the following members.

1. Dr. P.R. Nanayakkara

Affiliations: Consultant in Surgical Oncology, Teaching Hospital Jaffna, Sri Lanka

Email: pubudu_randima@hotmail.com

1. Dr.Sachith Aloka Abhayaratna

Affiliations: Senior Lecturer (Grade II) in Pharmacology, Faculty of Medicine, University of Colombo, Sri Lanka and Specialist in Endocrinology

Email: drsachith@yahoo.com

1. Dr. Shreenika De Silva Weliange

Affiliations: Senior Lecturer in Community Medicine, Faculty of Medicine, University of Colombo, Sri Lanka

Email: shreenika73@yahoo.co.uk

**Data Collection Tool (English)**

**Before administration of Radioactive iodine**

**Demographics**

1. Identification Number:
2. Age:
3. Gender: Male/ Female
4. Ethnicity: Sinhala/ Tamil/ Muslim/ Burgher/ Other -
5. Address:
6. Telephone Number:
7. Highest Education level:

**Medical history**

1. Year of diagnosis of thyroid cancer
2. Histology type:
3. Tumour Dimension:
4. Grading: well differenciated/ moderately differentiated/ poorly differentiated
5. Psammoma bodies: Yes/ No
6. Marked nuclear atypia: yes/ No
7. Tumour necrosis: Yes/ No
8. Vascular invasion: Yes/ No
9. Mitotic frequency: (Mean per high power field)
10. TNM Stage: T N M
11. Tumour invasion: None/ Thyroid capsule/ Muscle
12. Lymph node spread: None/ Intra nodal/ Extra nodal
13. Immunohistochemistry report:……………………………………
14. Post op Thyroglobulin levels (if available)/ Date
15. Post op thyroid stimulating hormone (TSH) levels (if available)/ Date
16. Surgery type:
17. Surgery date:
18. Radiation therapy: Yes/No Start date: End date:
19. Radioisotope scan findings: Residual tissue Yes / No Site: Neck / Other (Specify…………………………)
20. Dose of radioactive iodine to be given:

**Concurrent medications**

1. Give the list of comorbidities/ duration of illness and the medication

| Comorbidity | Duration of illness | Current treatment regime |
| --- | --- | --- |
|  |  |  |
|  |  |  |
|  |  |  |
|  |  |  |
|  |  |  |
|  |  |  |
|  |  |  |

**Baseline evaluation of symptoms and physical examination**

1. Height (cm):
2. Weight (kg):
3. Baseline symptoms and examination (with definition) CTCAE: Common terminology criteria for adverse effects version 4.0

| Symptom/ sign | Definition | Yes/ No | Duration if present | CTCAE grade if present |
| --- | --- | --- | --- | --- |
| Neck pain | A disorder characterized by marked discomfort sensation in the neck area |  |  |  |
| Neck swelling | A disorder characterized by marked enlargement in the neck area |  |  |  |
| Dysphagia | A disorder characterized by difficulty in swallowing |  |  |  |
| Dyspnoea | A disorder characterized by difficulty in breathing |  |  |  |
| Sialadenitis | A disorder characterized by an inflammatory process involving the salivary gland |  |  |  |
| Dry mouth | A disorder characterized by reduced salivary flow in the oral cavity |  |  |  |
| Oral mucositis | A disorder characterized by inflammation of the oral mucosal |  |  |  |
| Oral pain | A disorder characterized by a sensation of marked discomfort in the mouth, tongue or lip |  |  |  |
| Dysgeusia | A disorder characterized by abnormal sensual experience with the taste of foodstuffs |  |  |  |
| Nausea | A disorder characterized by a queasy sensation and/or the urge to vomit. |  |  |  |
| Vomiting | A disorder characterized by the reflexive act of ejecting the contents of the stomach through the mouth |  |  |  |
| Dyspepsia | A disorder characterized by an uncomfortable, often painful feeling in the stomach, resulting from impaired digestion. Symptoms include burning stomach, bloating, heartburn, nausea and vomiting |  |  |  |
| Xerophthalmia | A disorder characterized by dryness of the cornea and conjunctiva |  |  |  |
| Epiphoria | A disorder of excessive tearing in the eyes |  |  |  |
| Conjunctivitis | A disorder characterized by inflammation, to the conjunctiva of the eye |  |  |  |
| Infections within 2 weeks | A disorder characterized by an infectious process |  |  |  |
| Hyperglycaemia | A disorder characterized by laboratory test results that indicate an elevation in the concentration of blood sugar |  |  |  |
| Other 1 | Definition (CTCAE) |  |  |  |
| Other 2 |  |  |  |  |
| Other 3 |  |  |  |  |

**Performance status:**

1. ECOG performance status (Circle the correct number)

| Grade | ECOG performance status |
| --- | --- |
| 0 | Fully active, able to carry on all pre-disease performance without restriction |
| 1 | Restricted in physically strenuous activity but ambulatory and able to carry out work of a light or sedentary nature, e.g., light house work, office work |
| 2 | Ambulatory and capable of all selfcare but unable to carry out any work activities; up and about more than 50% of waking hours |
| 3 | Capable of only limited selfcare; confined to bed or chair more than 50% of waking hours |
| 4 | Completely disabled; cannot carry on any selfcare; totally confined to bed or chair |
| 5 | Dead |

**Baseline Quality of life (QOL) measurements**

1. EQ-5D-5L (UK English sample version**)**

Under each heading, please tick the **ONE** box that best describes your health **TODAY**

**MOBILITY**

I have no problems in walking about 

I have slight problems in walking about 

I have moderate problems in walking about 

I have severe problems in walking about 

I am unable to walk about 

**SELF-CARE**

I have no problems washing or dressing myself 

I have slight problems washing or dressing myself 

I have moderate problems washing or dressing myself 

I have severe problems washing or dressing myself 

I am unable to wash or dress myself 

**USUAL** **ACTIVITIES** *(e.g.* *work,* *study,* *housework,* *family* *or* *leisure* *activities)*

I have no problems doing my usual activities 

I have slight problems doing my usual activities 

I have moderate problems doing my usual activities 

I have severe problems doing my usual activities 

I am unable to do my usual activities 


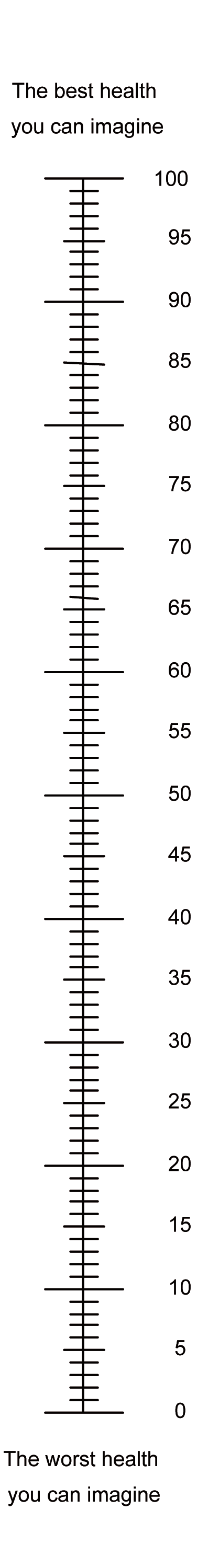
**PAIN** **/** **DISCOMFORT**

I have no pain or discomfort 

I have slight pain or discomfort 

I have moderate pain or discomfort 

I have severe pain or discomfort 

I have extreme pain or discomfort 

**ANXIETY** **/** **DEPRESSION**

I am not anxious or depressed 

I am slightly anxious or depressed 

I am moderately anxious or depressed 

I am severely anxious or depressed 

I am extremely anxious or depressed 


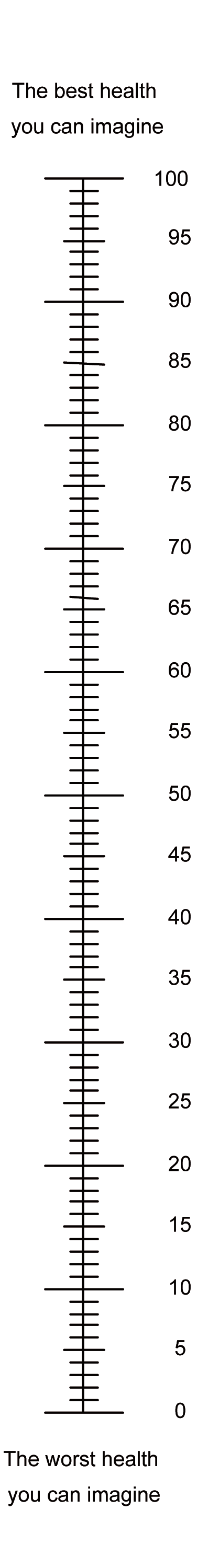
We would like to know how good or bad your health is **TODAY**.

This scale is numbered from **0** to **100**.

**100** means the best health you can imagine.

**0** means the worst health you can imagine.

Mark an **X** on the scale to indicate how your health is **TODAY**.

Now, please write the number you marked on the scale in the box below.

**YOUR** **HEALTH** **TODAY** =

**FACT-H&N (Version 4)**

|  | **PHYSICAL WELL-BEING** | **Not**  **at all** | **A little**  **bit** | **Some-**  **what** | **Quite**  **a bit** | **Very**  **much** |
| --- | --- | --- | --- | --- | --- | --- |
| GP1 | I have a lack of energy ....................................................... | 0 | 1 | 2 | 3 | 4 |
| GP2 | I have nausea ...................................................................... | 0 | 1 | 2 | 3 | 4 |
| GP3 | Because of my physical condition, I have trouble  meeting the needs of my family ........................... | 0 | 1 | 2 | 3 | 4 |
| GP4 | I have pain .......................................................................... | 0 | 1 | 2 | 3 | 4 |
| GP5 | I am bothered by side effects of treatment .................. | 0 | 1 | 2 | 3 | 4 |
| GP6 | I feel ill ............................................................................... | 0 | 1 | 2 | 3 | 4 |
| GP7 | I am forced to spend time in bed ........................................ | 0 | 1 | 2 | 3 | 4 |
|  | **SOCIAL/FAMILY WELL-BEING** |  |  |  |  |  |
| GS1 | I feel close to my friends ............................... | 0 | 1 | 2 | 3 | 4 |
| GS2 | I get emotional support from my family ..................... | 0 | 1 | 2 | 3 | 4 |
| GS3 | I get support from my friends........................ . | 0 | 1 | 2 | 3 | 4 |
| GS4 | My family has accepted my illness ................... | 0 | 1 | 2 | 3 | 4 |
| GS5 | I am satisfied with family communication about my  illness................................. | 0 | 1 | 2 | 3 | 4 |
| GS6 | I feel close to my partner (or the person who is my main support) ...................................................... | 0 | 1 | 2 | 3 | 4 |
|  | *Regardless of your current level of sexual activity, please answer the following question. If you prefer not to answer it, please mark this box and go to the next section.* |  | | | | |
| GS7 | I am satisfied with my sex life ...................................... | 0 | 1 | 2 | 3 | 4 |

Below is a list of statements that other people with your illness have said are important**. Please**

**circle** **or mark one number per line to indicate your response as it applies to the past 7 days.**

**FACT-H&N (Version 4)**

**Please circle or mark one number per line to indicate your response as it applies to the past 7 days.**

|  | **EMOTIONAL WELL-BEING** | **Not**  **at all** | **A little**  **bit** | **Some-**  **what** | **Quite**  **a bit** | **Very**  **much** |
| --- | --- | --- | --- | --- | --- | --- |
| GE1 | I feel sad .................................................. | 0 | 1 | 2 | 3 | 4 |
| GE2 | I am satisfied with how I am coping with my illness......... | 0 | 1 | 2 | 3 | 4 |
| GE3 | I am losing hope in the fight against my illness................. | 0 | 1 | 2 | 3 | 4 |
| GE4 | I feel nervous.............................. | 0 | 1 | 2 | 3 | 4 |
| GE5 | I worry about dying .................................... | 0 | 1 | 2 | 3 | 4 |
| GE6 | I worry that my condition will get worse ............ | 0 | 1 | 2 | 3 | 4 |

|  | **FUNCTIONAL WELL-BEING** | **Not**  **at all** | **A little**  **bit** | **Some-**  **what** | **Quite**  **a bit** | **Very**  **much** |
| --- | --- | --- | --- | --- | --- | --- |
| GF1 | I am able to work (include work at home) ............... | 0 | 1 | 2 | 3 | 4 |
| GF2 | My work (include work at home) is fulfilling........... | 0 | 1 | 2 | 3 | 4 |
| GF3 | I am able to enjoy life....................... | 0 | 1 | 2 | 3 | 4 |
| GF4 | I have accepted my illness....................... | 0 | 1 | 2 | 3 | 4 |
| GF5 | I am sleeping well ........................... | 0 | 1 | 2 | 3 | 4 |
| GF6 | I am enjoying the things I usually do for fun ........... | 0 | 1 | 2 | 3 | 4 |
| GF7 | I am content with the quality of my life right now... | 0 | 1 | 2 | 3 | 4 |

**FACT-H&N (Version 4)**

**Please circle or mark one number per line to indicate your response as it applies to the past 7 days.**

|  | **ADDITIONAL CONCERNS** | **Not at**  **all** | **A little**  **bit** | **Some-**  **what** | **Quite**  **a bit** | **Very**  **much** |
| --- | --- | --- | --- | --- | --- | --- |
| H&N1 | I am able to eat the foods that I like ........... | 0 | 1 | 2 | 3 | 4 |
| H&N1 | My mouth is dry ............................ | 0 | 1 | 2 | 3 | 4 |
| H&N1 | I have trouble breathing ............................. | 0 | 1 | 2 | 3 | 4 |
| H&N1 | My voice has its usual quality and strength ... | 0 | 1 | 2 | 3 | 4 |
| H&N1 | I am able to eat as much food as I want ......... | 0 | 1 | 2 | 3 | 4 |
| H&N1 | I am unhappy with how my face and neck look… | 0 | 1 | 2 | 3 | 4 |
| H&N1 | I can swallow naturally and easily ............. | 0 | 1 | 2 | 3 | 4 |
| H&N1 | I smoke cigarettes or other tobacco products..... | 0 | 1 | 2 | 3 | 4 |
| H&N1 | I drink alcohol (e.g. beer, wine, etc.).......... | 0 | 1 | 2 | 3 | 4 |
| H&N1 | I am able to communicate with others ........... | 0 | 1 | 2 | 3 | 4 |
| H&N1 | I can eat solid foods............... | 0 | 1 | 2 | 3 | 4 |
| H&N1 | I have pain in my mouth, throat or neck ......... | 0 | 1 | 2 | 3 | 4 |

1. **Patient reported outcomes assessment**
2. **PRO-CTCAE™ Symptom Term:** NECK PAIN

a. In the last 7 days, what was the SEVERITY of your NECK PAIN at its WORST?

| Ο None | Ο Mild | Ο Moderate | Ο Severe | Ο Very severe |
| --- | --- | --- | --- | --- |

1. **PRO-CTCAE™ Symptom Term:** NECK SWELLING

a. In the last 7 days, what was the SEVERITY of your NECK SWELLING at its WORST?

| Ο None | Ο Mild | Ο Moderate | Ο Severe | Ο Very severe |
| --- | --- | --- | --- | --- |

1. **PRO-CTCAE™ Symptom Term:** Difficulty swallowing

a. In the last 7 days, what was the SEVERITY of your DIFFICLTY SWALLOWING at its WORST?

| Ο None | Ο Mild | Ο Moderate | Ο Severe | Ο Very severe |
| --- | --- | --- | --- | --- |

1. **PRO-CTCAE™ Symptom Term:** Shortness of breath

a. In the last 7 days, what was the SEVERITY of your SHORTNESS OF BREATH at its WORST?

| Ο None | Ο Mild | Ο Moderate | Ο Severe | Ο Very severe |
| --- | --- | --- | --- | --- |

b. In the last 7 days, how much did your SHORTNESS OF BREATH INTERFERE with your usual or daily activities?

| Ο Not at all | Ο A little bit | Ο Somewhat | Ο Quite a bit | Ο Very much |
| --- | --- | --- | --- | --- |

1. **PRO-CTCAE™ Symptom Term:** PAIN OVER SALIVARY GLANDS

a. In the last 7 days, what was the SEVERITY of your PAIN OVER SALIVARY GLANDS at its WORST?

| Ο None | Ο Mild | Ο Moderate | Ο Severe | Ο Very severe |
| --- | --- | --- | --- | --- |

1. **PRO-CTCAE™ Symptom Term:** Dry mouth

a. In the last 7 days, what was the SEVERITY of your DRY MOUTH at its WORST?

| Ο None | Ο Mild | Ο Moderate | Ο Severe | Ο Very severe |
| --- | --- | --- | --- | --- |

1. **PRO-CTCAE™ Symptom Term:** Mouth/throat sores

a. In the last 7 days, what was the SEVERITY of your MOUTH OR THROAT SORES at their WORST?

| Ο None | Ο Mild | Ο Moderate | Ο Severe | Ο Very severe |
| --- | --- | --- | --- | --- |

b. In the last 7 days, how much did MOUTH OR THROAT SORES INTERFERE with your usual or daily activities?

| Ο Not at all | Ο A little bit | Ο Somewhat | Ο Quite a bit | Ο Very much |
| --- | --- | --- | --- | --- |

1. **PRO-CTCAE™ Symptom Term:** ORAL PAIN

a. In the last 7 days, what was the SEVERITY of your ORAL PAIN at its WORST?

| Ο None | Ο Mild | Ο Moderate | Ο Severe | Ο Very severe |
| --- | --- | --- | --- | --- |

1. **PRO-CTCAE™ Symptom Term:** Taste changes

a. In the last 7 days, what was the SEVERITY of your PROBLEMS WITH TASTING FOOD OR DRINK at their

WORST?

| Ο None | Ο Mild | Ο Moderate | Ο Severe | Ο Very severe |
| --- | --- | --- | --- | --- |

1. **PRO-CTCAE™ Symptom Term:** Nausea

a. In the last 7 days, how OFTEN did you have NAUSEA?

| Ο Never | Ο Rarely | Ο Occasionally | Ο Frequently | Ο Almost constantly |
| --- | --- | --- | --- | --- |

b. In the last 7 days, what was the SEVERITY of your NAUSEA at its WORST?

| Ο None | Ο Mild | Ο Moderate | Ο Severe | Ο Very severe |
| --- | --- | --- | --- | --- |

1. **PRO-CTCAE™ Symptom Term:** Vomiting

a. In the last 7 days, how OFTEN did you have VOMITING?

| Ο Never | Ο Rarely | Ο Occasionally | Ο Frequently | Ο Almost constantly |
| --- | --- | --- | --- | --- |

b. In the last 7 days, what was the SEVERITY of your VOMITING at its WORST?

| Ο None | Ο Mild | Ο Moderate | Ο Severe | Ο Very severe |
| --- | --- | --- | --- | --- |

1. **PRO-CTCAE™ Symptom Term:** Heartburn

a. In the last 7 days, how OFTEN did you have HEARTBURN?

| Ο Never | Ο Rarely | Ο Occasionally | Ο Frequently | Ο Almost constantly |
| --- | --- | --- | --- | --- |

b. In the last 7 days, what was the SEVERITY of your HEARTBURN at its WORST?

| Ο None | Ο Mild | Ο Moderate | Ο Severe | Ο Very severe |
| --- | --- | --- | --- | --- |

1. **PRO-CTCAE™ Symptom Term:** Abdominal pain

a. In the last 7 days, how OFTEN did you have PAIN IN THE ABDOMEN (BELLY AREA)?

| Ο Never | Ο Rarely | Ο Occasionally | Ο Frequently | Ο Almost constantly |
| --- | --- | --- | --- | --- |

b. In the last 7 days, what was the SEVERITY of your PAIN IN THE ABDOMEN (BELLY AREA) at its WORST?

| Ο None | Ο Mild | Ο Moderate | Ο Severe | Ο Very severe |
| --- | --- | --- | --- | --- |

c. In the last 7 days, how much did PAIN IN THE ABDOMEN (BELLY AREA) INTERFERE with your usual or daily activities?

| Ο Not at all | Ο A little bit | Ο Somewhat | Ο Quite a bit | Ο Very much |
| --- | --- | --- | --- | --- |

1. **PRO-CTCAE™ Symptom Term:** Dry eyes

a. In the last 7 days, what was the SEVERITY of your DRY EYES at its WORST?

| Ο None | Ο Mild | Ο Moderate | Ο Severe | Ο Very severe |
| --- | --- | --- | --- | --- |

1. **PRO-CTCAE™ Symptom Term:** Watery eyes

a. In the last 7 days, what was the SEVERITY of your WATERY EYES (TEARING) at their WORST?

| Ο None | Ο Mild | Ο Moderate | Ο Severe | Ο Very severe |
| --- | --- | --- | --- | --- |

b. In the last 7 days, how much did WATERY EYES (TEARING) INTERFERE with your usual or daily activities?

| Ο Not at all | Ο A little bit | Ο Somewhat | Ο Quite a bit | Ο Very much |
| --- | --- | --- | --- | --- |

1. **PRO-CTCAE™ Symptom Term:** Painful red eyes

a. In the last 7 days, what was the SEVERITY of your PAINFUL RED EYES at its WORST?

| Ο None | Ο Mild | Ο Moderate | Ο Severe | Ο Very severe |
| --- | --- | --- | --- | --- |

**At follow up visit at 2 weeks**

| Symptom/ sign | Yes/ No | Onset | Duration | CTCAE grade | Trend | Treatment | Response to treatment |
| --- | --- | --- | --- | --- | --- | --- | --- |
| Neck pain |  |  |  |  |  |  |  |
| Neck swelling |  |  |  |  |  |  |  |
| Dysphagia |  |  |  |  |  |  |  |
| Dyspnoea |  |  |  |  |  |  |  |
| Sialadenitis |  |  |  |  |  |  |  |
| Dry mouth |  |  |  |  |  |  |  |
| Oral mucositis |  |  |  |  |  |  |  |
| Oral pain |  |  |  |  |  |  |  |
| Dysgeusia |  |  |  |  |  |  |  |
| Nausea |  |  |  |  |  |  |  |
| Vomiting |  |  |  |  |  |  |  |
| Dyspepsia |  |  |  |  |  |  |  |
| Xerophthalmia |  |  |  |  |  |  |  |
| Epiphoria |  |  |  |  |  |  |  |
| Conjunctivitis |  |  |  |  |  |  |  |
| Infections within 2 weeks |  |  |  |  |  |  |  |
| Hyperglycaemia |  |  |  |  |  |  |  |
| Other 1 |  |  |  |  |  |  |  |
| Other 2 |  |  |  |  |  |  |  |
| Other 3 |  |  |  |  |  |  |  |

Trend: A: Incresing severity, B: Decreasing severity, C: Static D: Fluctuating severity

Response to treatment: A: Good response, B: Some response, C: No response

**Performance status:**

1. ECOG performance status (Circle the correct number)

| Grade | ECOG performance status |
| --- | --- |
| 0 | Fully active, able to carry on all pre-disease performance without restriction |
| 1 | Restricted in physically strenuous activity but ambulatory and able to carry out work of a light or sedentary nature, e.g., light house work, office work |
| 2 | Ambulatory and capable of all selfcare but unable to carry out any work activities; up and about more than 50% of waking hours |
| 3 | Capable of only limited selfcare; confined to bed or chair more than 50% of waking hours |
| 4 | Completely disabled; cannot carry on any selfcare; totally confined to bed or chair |
| 5 | Dead |

**Quality of life (QOL) measurements at 2 weeks**

1. EQ-5D-5L (UK English sample version)

Under each heading, please tick the **ONE** box that best describes your health **TODAY**

**MOBILITY**

I have no problems in walking about 

I have slight problems in walking about 

I have moderate problems in walking about 

I have severe problems in walking about 

I am unable to walk about 

**SELF-CARE**

I have no problems washing or dressing myself 

I have slight problems washing or dressing myself 

I have moderate problems washing or dressing myself 

I have severe problems washing or dressing myself 

I am unable to wash or dress myself 

**USUAL** **ACTIVITIES** *(e.g.* *work,* *study,* *housework,* *family* *or* *leisure* *activities)*

I have no problems doing my usual activities 

I have slight problems doing my usual activities 

I have moderate problems doing my usual activities 

I have severe problems doing my usual activities 

I am unable to do my usual activities 


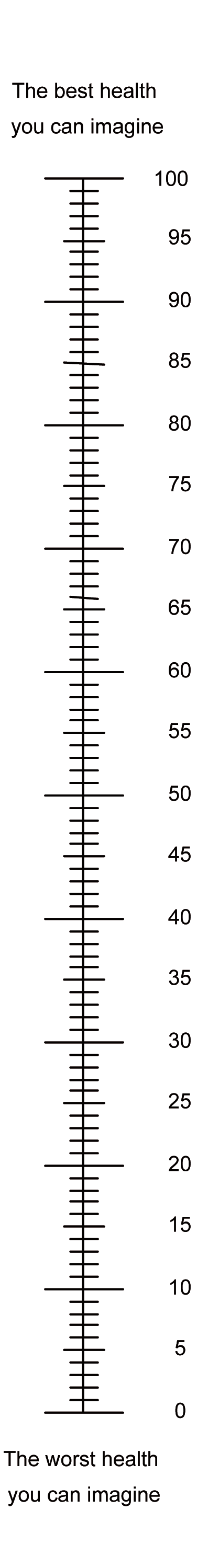

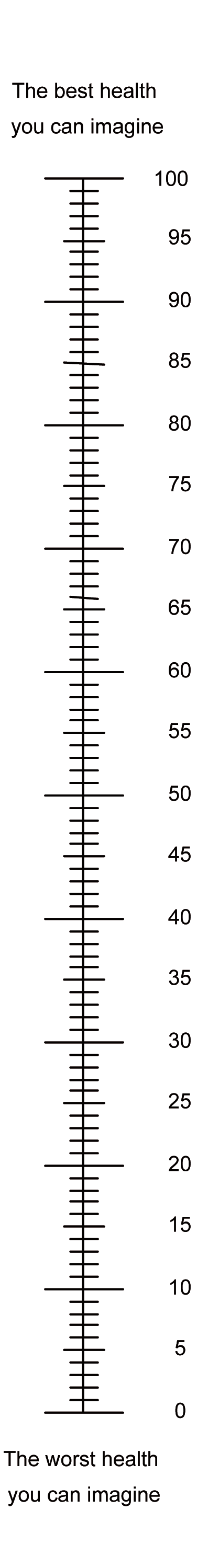
**PAIN** **/** **DISCOMFORT**

I have no pain or discomfort 

I have slight pain or discomfort 

I have moderate pain or discomfort 

I have severe pain or discomfort 

I have extreme pain or discomfort 

**ANXIETY** **/** **DEPRESSION**

I am not anxious or depressed 

I am slightly anxious or depressed 

I am moderately anxious or depressed 

I am severely anxious or depressed 

I am extremely anxious or depressed 

We would like to know how good or bad your health is **TODAY**.

This scale is numbered from **0** to **100**.

**100** means the best health you can imagine.

**0** means the worst health you can imagine.

Mark an **X** on the scale to indicate how your health is **TODAY**.

Now, please write the number you marked on the scale in the box below.

**YOUR** **HEALTH** **TODAY** =

**FACT-H&N (Version 4)**

|  | **PHYSICAL WELL-BEING** | **Not**  **at all** | **A little**  **bit** | **Some-**  **what** | **Quite**  **a bit** | **Very**  **much** |
| --- | --- | --- | --- | --- | --- | --- |
| GP1 | I have a lack of energy ....................................................... | 0 | 1 | 2 | 3 | 4 |
| GP2 | I have nausea ...................................................................... | 0 | 1 | 2 | 3 | 4 |
| GP3 | Because of my physical condition, I have trouble  meeting the needs of my family ........................... | 0 | 1 | 2 | 3 | 4 |
| GP4 | I have pain .......................................................................... | 0 | 1 | 2 | 3 | 4 |
| GP5 | I am bothered by side effects of treatment .................. | 0 | 1 | 2 | 3 | 4 |
| GP6 | I feel ill ............................................................................... | 0 | 1 | 2 | 3 | 4 |
| GP7 | I am forced to spend time in bed ........................................ | 0 | 1 | 2 | 3 | 4 |
|  | **SOCIAL/FAMILY WELL-BEING** |  |  |  |  |  |
| GS1 | I feel close to my friends ............................... | 0 | 1 | 2 | 3 | 4 |
| GS2 | I get emotional support from my family ..................... | 0 | 1 | 2 | 3 | 4 |
| GS3 | I get support from my friends........................ . | 0 | 1 | 2 | 3 | 4 |
| GS4 | My family has accepted my illness ................... | 0 | 1 | 2 | 3 | 4 |
| GS5 | I am satisfied with family communication about my  illness................................. | 0 | 1 | 2 | 3 | 4 |
| GS6 | I feel close to my partner (or the person who is my main support) ...................................................... | 0 | 1 | 2 | 3 | 4 |
|  | *Regardless of your current level of sexual activity, please answer the following question. If you prefer not to answer it, please mark this box and go to the next section.* |  | | | | |
| GS7 | I am satisfied with my sex life ...................................... | 0 | 1 | 2 | 3 | 4 |

Below is a list of statements that other people with your illness have said are important**. Please**

**circle** **or mark one number per line to indicate your response as it applies to the past 7 days.**

**FACT-H&N (Version 4)**

**Please circle or mark one number per line to indicate your response as it applies to the past 7 days.**

|  | **EMOTIONAL WELL-BEING** | **Not**  **at all** | **A little**  **bit** | **Some-**  **what** | **Quite**  **a bit** | **Very**  **much** |
| --- | --- | --- | --- | --- | --- | --- |
| GE1 | I feel sad .................................................. | 0 | 1 | 2 | 3 | 4 |
| GE2 | I am satisfied with how I am coping with my illness......... | 0 | 1 | 2 | 3 | 4 |
| GE3 | I am losing hope in the fight against my illness................. | 0 | 1 | 2 | 3 | 4 |
| GE4 | I feel nervous.............................. | 0 | 1 | 2 | 3 | 4 |
| GE5 | I worry about dying .................................... | 0 | 1 | 2 | 3 | 4 |
| GE6 | I worry that my condition will get worse ............ | 0 | 1 | 2 | 3 | 4 |

|  | **FUNCTIONAL WELL-BEING** | **Not**  **at all** | **A little**  **bit** | **Some-**  **what** | **Quite**  **a bit** | **Very**  **much** |
| --- | --- | --- | --- | --- | --- | --- |
| GF1 | I am able to work (include work at home) ............... | 0 | 1 | 2 | 3 | 4 |
| GF2 | My work (include work at home) is fulfilling........... | 0 | 1 | 2 | 3 | 4 |
| GF3 | I am able to enjoy life....................... | 0 | 1 | 2 | 3 | 4 |
| GF4 | I have accepted my illness....................... | 0 | 1 | 2 | 3 | 4 |
| GF5 | I am sleeping well ........................... | 0 | 1 | 2 | 3 | 4 |
| GF6 | I am enjoying the things I usually do for fun ........... | 0 | 1 | 2 | 3 | 4 |
| GF7 | I am content with the quality of my life right now... | 0 | 1 | 2 | 3 | 4 |

**FACT-H&N (Version 4)**

**Please circle or mark one number per line to indicate your response as it applies to the past 7 days.**

|  | **ADDITIONAL CONCERNS** | **Not at**  **all** | **A little**  **bit** | **Some-**  **what** | **Quite**  **a bit** | **Very**  **much** |
| --- | --- | --- | --- | --- | --- | --- |
| H&N1 | I am able to eat the foods that I like ........... | 0 | 1 | 2 | 3 | 4 |
| H&N1 | My mouth is dry ............................ | 0 | 1 | 2 | 3 | 4 |
| H&N1 | I have trouble breathing ............................. | 0 | 1 | 2 | 3 | 4 |
| H&N1 | My voice has its usual quality and strength ... | 0 | 1 | 2 | 3 | 4 |
| H&N1 | I am able to eat as much food as I want ......... | 0 | 1 | 2 | 3 | 4 |
| H&N1 | I am unhappy with how my face and neck look… | 0 | 1 | 2 | 3 | 4 |
| H&N1 | I can swallow naturally and easily ............. | 0 | 1 | 2 | 3 | 4 |
| H&N1 | I smoke cigarettes or other tobacco products..... | 0 | 1 | 2 | 3 | 4 |
| H&N1 | I drink alcohol (e.g. beer, wine, etc.).......... | 0 | 1 | 2 | 3 | 4 |
| H&N1 | I am able to communicate with others ........... | 0 | 1 | 2 | 3 | 4 |
| H&N1 | I can eat solid foods............... | 0 | 1 | 2 | 3 | 4 |
| H&N1 | I have pain in my mouth, throat or neck ......... | 0 | 1 | 2 | 3 | 4 |

**Patient reported outcomes assessment**

1. **PRO-CTCAE™ Symptom Term:** NECK PAIN

a. In the last 7 days, what was the SEVERITY of your NECK PAIN at its WORST?

| Ο None | Ο Mild | Ο Moderate | Ο Severe | Ο Very severe |
| --- | --- | --- | --- | --- |

1. **PRO-CTCAE™ Symptom Term:** NECK SWELLING

a. In the last 7 days, what was the SEVERITY of your NECK SWELLING at its WORST?

| Ο None | Ο Mild | Ο Moderate | Ο Severe | Ο Very severe |
| --- | --- | --- | --- | --- |

1. **PRO-CTCAE™ Symptom Term:** Difficulty swallowing

a. In the last 7 days, what was the SEVERITY of your DIFFICLTY SWALLOWING at its WORST?

| Ο None | Ο Mild | Ο Moderate | Ο Severe | Ο Very severe |
| --- | --- | --- | --- | --- |

1. **PRO-CTCAE™ Symptom Term:** Shortness of breath

a. In the last 7 days, what was the SEVERITY of your SHORTNESS OF BREATH at its WORST?

| Ο None | Ο Mild | Ο Moderate | Ο Severe | Ο Very severe |
| --- | --- | --- | --- | --- |

b. In the last 7 days, how much did your SHORTNESS OF BREATH INTERFERE with your usual or daily activities?

| Ο Not at all | Ο A little bit | Ο Somewhat | Ο Quite a bit | Ο Very much |
| --- | --- | --- | --- | --- |

1. **PRO-CTCAE™ Symptom Term:** PAIN OVER SALIVARY GLANDS

a. In the last 7 days, what was the SEVERITY of your PAIN OVER SALIVARY GLANDS at its WORST?

| Ο None | Ο Mild | Ο Moderate | Ο Severe | Ο Very severe |
| --- | --- | --- | --- | --- |

1. **PRO-CTCAE™ Symptom Term:** Dry mouth

a. In the last 7 days, what was the SEVERITY of your DRY MOUTH at its WORST?

| Ο None | Ο Mild | Ο Moderate | Ο Severe | Ο Very severe |
| --- | --- | --- | --- | --- |

1. **PRO-CTCAE™ Symptom Term:** Mouth/throat sores

a. In the last 7 days, what was the SEVERITY of your MOUTH OR THROAT SORES at their WORST?

| Ο None | Ο Mild | Ο Moderate | Ο Severe | Ο Very severe |
| --- | --- | --- | --- | --- |

b. In the last 7 days, how much did MOUTH OR THROAT SORES INTERFERE with your usual or daily activities?

| Ο Not at all | Ο A little bit | Ο Somewhat | Ο Quite a bit | Ο Very much |
| --- | --- | --- | --- | --- |

1. **PRO-CTCAE™ Symptom Term:** ORAL PAIN

a. In the last 7 days, what was the SEVERITY of your ORAL PAIN at its WORST?

| Ο None | Ο Mild | Ο Moderate | Ο Severe | Ο Very severe |
| --- | --- | --- | --- | --- |

1. **PRO-CTCAE™ Symptom Term:** Taste changes

a. In the last 7 days, what was the SEVERITY of your PROBLEMS WITH TASTING FOOD OR DRINK at their WORST?

| Ο None | Ο Mild | Ο Moderate | Ο Severe | Ο Very severe |
| --- | --- | --- | --- | --- |

1. **PRO-CTCAE™ Symptom Term:** Nausea

a. In the last 7 days, how OFTEN did you have NAUSEA?

| Ο Never | Ο Rarely | Ο Occasionally | Ο Frequently | Ο Almost constantly |
| --- | --- | --- | --- | --- |

b. In the last 7 days, what was the SEVERITY of your NAUSEA at its WORST?

| Ο None | Ο Mild | Ο Moderate | Ο Severe | Ο Very severe |
| --- | --- | --- | --- | --- |

1. **PRO-CTCAE™ Symptom Term:** Vomiting

a. In the last 7 days, how OFTEN did you have VOMITING?

| Ο Never | Ο Rarely | Ο Occasionally | Ο Frequently | Ο Almost constantly |
| --- | --- | --- | --- | --- |

b. In the last 7 days, what was the SEVERITY of your VOMITING at its WORST?

| Ο None | Ο Mild | Ο Moderate | Ο Severe | Ο Very severe |
| --- | --- | --- | --- | --- |

1. **PRO-CTCAE™ Symptom Term:** Heartburn

a. In the last 7 days, how OFTEN did you have HEARTBURN?

| Ο Never | Ο Rarely | Ο Occasionally | Ο Frequently | Ο Almost constantly |
| --- | --- | --- | --- | --- |

b. In the last 7 days, what was the SEVERITY of your HEARTBURN at its WORST?

| Ο None | Ο Mild | Ο Moderate | Ο Severe | Ο Very severe |
| --- | --- | --- | --- | --- |

1. **PRO-CTCAE™ Symptom Term:** Abdominal pain

a. In the last 7 days, how OFTEN did you have PAIN IN THE ABDOMEN (BELLY AREA)?

| Ο Never | Ο Rarely | Ο Occasionally | Ο Frequently | Ο Almost constantly |
| --- | --- | --- | --- | --- |

b. In the last 7 days, what was the SEVERITY of your PAIN IN THE ABDOMEN (BELLY AREA) at its WORST?

| Ο None | Ο Mild | Ο Moderate | Ο Severe | Ο Very severe |
| --- | --- | --- | --- | --- |

c. In the last 7 days, how much did PAIN IN THE ABDOMEN (BELLY AREA) INTERFERE with your usual or daily activities?

| Ο Not at all | Ο A little bit | Ο Somewhat | Ο Quite a bit | Ο Very much |
| --- | --- | --- | --- | --- |

1. **PRO-CTCAE™ Symptom Term:** Dry eyes

a. In the last 7 days, what was the SEVERITY of your DRY EYES at its WORST?

| Ο None | Ο Mild | Ο Moderate | Ο Severe | Ο Very severe |
| --- | --- | --- | --- | --- |

1. **PRO-CTCAE™ Symptom Term:** Watery eyes

a. In the last 7 days, what was the SEVERITY of your WATERY EYES (TEARING) at their WORST?

| Ο None | Ο Mild | Ο Moderate | Ο Severe | Ο Very severe |
| --- | --- | --- | --- | --- |

b. In the last 7 days, how much did WATERY EYES (TEARING) INTERFERE with your usual or daily activities?

| Ο Not at all | Ο A little bit | Ο Somewhat | Ο Quite a bit | Ο Very much |
| --- | --- | --- | --- | --- |

1. **PRO-CTCAE™ Symptom Term:** Painful red eyes

a. In the last 7 days, what was the SEVERITY of your PAINFUL RED EYES at its WORST?

| Ο None | Ο Mild | Ο Moderate | Ο Severe | Ο Very severe |
| --- | --- | --- | --- | --- |

**At follow up visit at 3 months**

| Symptom/ sign | Yes/ No | Onset | Duration | CTCAE grade | Trend | Treatment | Response to treatment |
| --- | --- | --- | --- | --- | --- | --- | --- |
| Neck pain |  |  |  |  |  |  |  |
| Neck swelling |  |  |  |  |  |  |  |
| Dysphagia |  |  |  |  |  |  |  |
| Dyspnoea |  |  |  |  |  |  |  |
| Sialadenitis |  |  |  |  |  |  |  |
| Dry mouth |  |  |  |  |  |  |  |
| Oral mucositis |  |  |  |  |  |  |  |
| Oral pain |  |  |  |  |  |  |  |
| Dysgeusia |  |  |  |  |  |  |  |
| Nausea |  |  |  |  |  |  |  |
| Vomiting |  |  |  |  |  |  |  |
| Dyspepsia |  |  |  |  |  |  |  |
| Xerophthalmia |  |  |  |  |  |  |  |
| Epiphoria |  |  |  |  |  |  |  |
| Conjunctivitis |  |  |  |  |  |  |  |
| Infections within 2 weeks |  |  |  |  |  |  |  |
| Hyperglycaemia |  |  |  |  |  |  |  |
| Other 1 |  |  |  |  |  |  |  |
| Other 2 |  |  |  |  |  |  |  |
| Other 3 |  |  |  |  |  |  |  |

Trend: A: Incresing severity, B: Decreasing severity, C: Static D: Fluctuating severity

Response to treatment: A: Good response, B: Some response, C: No response

**Performance status:**

1. ECOG performance status (Circle the correct number)

| Grade | ECOG performance status |
| --- | --- |
| 0 | Fully active, able to carry on all pre-disease performance without restriction |
| 1 | Restricted in physically strenuous activity but ambulatory and able to carry out work of a light or sedentary nature, e.g., light house work, office work |
| 2 | Ambulatory and capable of all selfcare but unable to carry out any work activities; up and about more than 50% of waking hours |
| 3 | Capable of only limited selfcare; confined to bed or chair more than 50% of waking hours |
| 4 | Completely disabled; cannot carry on any selfcare; totally confined to bed or chair |
| 5 | Dead |

**Quality of life (QOL) measurements at 2 weeks**

1. EQ-5D-5L (UK English sample version)

Under each heading, please tick the **ONE** box that best describes your health **TODAY**

**MOBILITY**

I have no problems in walking about 

I have slight problems in walking about 

I have moderate problems in walking about 

I have severe problems in walking about 

I am unable to walk about 

**SELF-CARE**

I have no problems washing or dressing myself 

I have slight problems washing or dressing myself 

I have moderate problems washing or dressing myself 

I have severe problems washing or dressing myself 

I am unable to wash or dress myself 

**USUAL** **ACTIVITIES** *(e.g.* *work,* *study,* *housework,* *family* *or* *leisure* *activities)*

I have no problems doing my usual activities 

I have slight problems doing my usual activities 

I have moderate problems doing my usual activities 

I have severe problems doing my usual activities 

I am unable to do my usual activities 


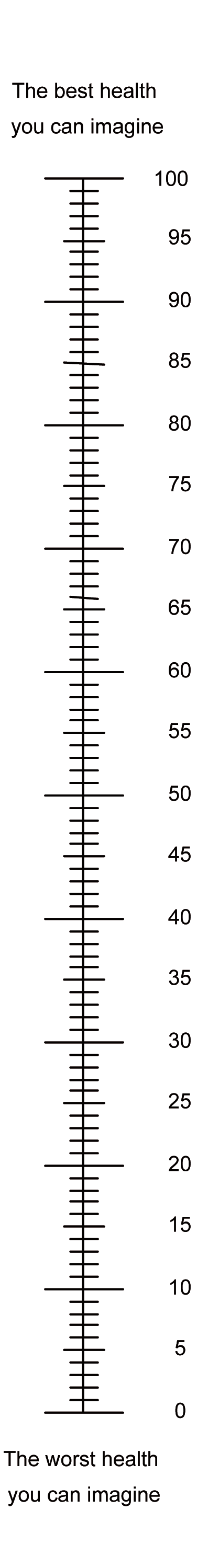
**PAIN** **/** **DISCOMFORT**

I have no pain or discomfort 

I have slight pain or discomfort 

I have moderate pain or discomfort 

I have severe pain or discomfort 

I have extreme pain or discomfort 

**ANXIETY** **/** **DEPRESSION**

I am not anxious or depressed 

I am slightly anxious or depressed 

I am moderately anxious or depressed 

I am severely anxious or depressed 

I am extremely anxious or depressed 


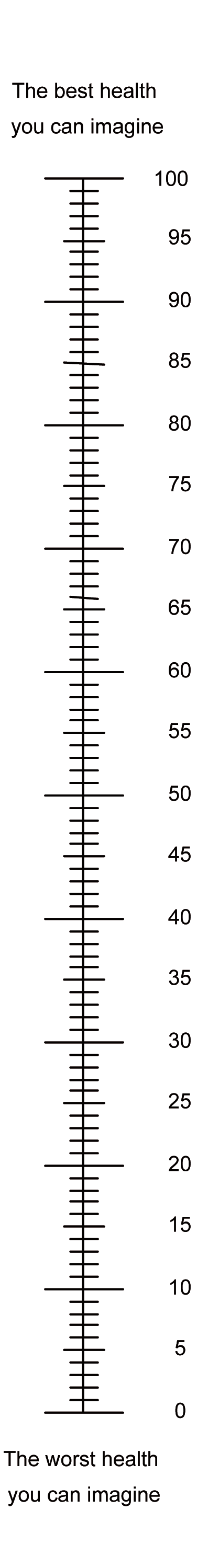
We would like to know how good or bad your health is **TODAY**.

This scale is numbered from **0** to **100**.

**100** means the best health you can imagine.

**0** means the worst health you can imagine.

Mark an **X** on the scale to indicate how your health is **TODAY**.

Now, please write the number you marked on the scale in the box below.

**YOUR** **HEALTH** **TODAY** =

**FACT-H&N (Version 4)**

|  | **PHYSICAL WELL-BEING** | **Not**  **at all** | **A little**  **bit** | **Some-**  **what** | **Quite**  **a bit** | **Very**  **much** |
| --- | --- | --- | --- | --- | --- | --- |
| GP1 | I have a lack of energy ....................................................... | 0 | 1 | 2 | 3 | 4 |
| GP2 | I have nausea ...................................................................... | 0 | 1 | 2 | 3 | 4 |
| GP3 | Because of my physical condition, I have trouble  meeting the needs of my family ........................... | 0 | 1 | 2 | 3 | 4 |
| GP4 | I have pain .......................................................................... | 0 | 1 | 2 | 3 | 4 |
| GP5 | I am bothered by side effects of treatment .................. | 0 | 1 | 2 | 3 | 4 |
| GP6 | I feel ill ............................................................................... | 0 | 1 | 2 | 3 | 4 |
| GP7 | I am forced to spend time in bed ........................................ | 0 | 1 | 2 | 3 | 4 |
|  | **SOCIAL/FAMILY WELL-BEING** |  |  |  |  |  |
| GS1 | I feel close to my friends ............................... | 0 | 1 | 2 | 3 | 4 |
| GS2 | I get emotional support from my family ..................... | 0 | 1 | 2 | 3 | 4 |
| GS3 | I get support from my friends........................ . | 0 | 1 | 2 | 3 | 4 |
| GS4 | My family has accepted my illness ................... | 0 | 1 | 2 | 3 | 4 |
| GS5 | I am satisfied with family communication about my  illness................................. | 0 | 1 | 2 | 3 | 4 |
| GS6 | I feel close to my partner (or the person who is my main support) ...................................................... | 0 | 1 | 2 | 3 | 4 |
|  | *Regardless of your current level of sexual activity, please answer the following question. If you prefer not to answer it, please mark this box and go to the next section.* |  | | | | |
| GS7 | I am satisfied with my sex life ...................................... | 0 | 1 | 2 | 3 | 4 |

Below is a list of statements that other people with your illness have said are important**. Please**

**circle** **or mark one number per line to indicate your response as it applies to the past 7 days.**

**FACT-H&N (Version 4)**

**Please circle or mark one number per line to indicate your response as it applies to the past 7 days.**

|  | **EMOTIONAL WELL-BEING** | **Not**  **at all** | **A little**  **bit** | **Some-**  **what** | **Quite**  **a bit** | **Very**  **much** |
| --- | --- | --- | --- | --- | --- | --- |
| GE1 | I feel sad .................................................. | 0 | 1 | 2 | 3 | 4 |
| GE2 | I am satisfied with how I am coping with my illness......... | 0 | 1 | 2 | 3 | 4 |
| GE3 | I am losing hope in the fight against my illness................. | 0 | 1 | 2 | 3 | 4 |
| GE4 | I feel nervous.............................. | 0 | 1 | 2 | 3 | 4 |
| GE5 | I worry about dying .................................... | 0 | 1 | 2 | 3 | 4 |
| GE6 | I worry that my condition will get worse ............ | 0 | 1 | 2 | 3 | 4 |

|  | **FUNCTIONAL WELL-BEING** | **Not**  **at all** | **A little**  **bit** | **Some-**  **what** | **Quite**  **a bit** | **Very**  **much** |
| --- | --- | --- | --- | --- | --- | --- |
| GF1 | I am able to work (include work at home) ............... | 0 | 1 | 2 | 3 | 4 |
| GF2 | My work (include work at home) is fulfilling........... | 0 | 1 | 2 | 3 | 4 |
| GF3 | I am able to enjoy life....................... | 0 | 1 | 2 | 3 | 4 |
| GF4 | I have accepted my illness....................... | 0 | 1 | 2 | 3 | 4 |
| GF5 | I am sleeping well ........................... | 0 | 1 | 2 | 3 | 4 |
| GF6 | I am enjoying the things I usually do for fun ........... | 0 | 1 | 2 | 3 | 4 |
| GF7 | I am content with the quality of my life right now... | 0 | 1 | 2 | 3 | 4 |

**FACT-H&N (Version 4)**

**Please circle or mark one number per line to indicate your response as it applies to the past 7 days.**

|  | **ADDITIONAL CONCERNS** | **Not at**  **all** | **A little**  **bit** | **Some-**  **what** | **Quite**  **a bit** | **Very**  **much** |
| --- | --- | --- | --- | --- | --- | --- |
| H&N1 | I am able to eat the foods that I like ........... | 0 | 1 | 2 | 3 | 4 |
| H&N1 | My mouth is dry ............................ | 0 | 1 | 2 | 3 | 4 |
| H&N1 | I have trouble breathing ............................. | 0 | 1 | 2 | 3 | 4 |
| H&N1 | My voice has its usual quality and strength ... | 0 | 1 | 2 | 3 | 4 |
| H&N1 | I am able to eat as much food as I want ......... | 0 | 1 | 2 | 3 | 4 |
| H&N1 | I am unhappy with how my face and neck look… | 0 | 1 | 2 | 3 | 4 |
| H&N1 | I can swallow naturally and easily ............. | 0 | 1 | 2 | 3 | 4 |
| H&N1 | I smoke cigarettes or other tobacco products..... | 0 | 1 | 2 | 3 | 4 |
| H&N1 | I drink alcohol (e.g. beer, wine, etc.).......... | 0 | 1 | 2 | 3 | 4 |
| H&N1 | I am able to communicate with others ........... | 0 | 1 | 2 | 3 | 4 |
| H&N1 | I can eat solid foods............... | 0 | 1 | 2 | 3 | 4 |
| H&N1 | I have pain in my mouth, throat or neck ......... | 0 | 1 | 2 | 3 | 4 |

**Patient reported outcomes assessment**

1. **PRO-CTCAE™ Symptom Term:** NECK PAIN

a. In the last 7 days, what was the SEVERITY of your NECK PAIN at its WORST?

| Ο None | Ο Mild | Ο Moderate | Ο Severe | Ο Very severe |
| --- | --- | --- | --- | --- |

1. **PRO-CTCAE™ Symptom Term:** NECK SWELLING

a. In the last 7 days, what was the SEVERITY of your NECK SWELLING at its WORST?

| Ο None | Ο Mild | Ο Moderate | Ο Severe | Ο Very severe |
| --- | --- | --- | --- | --- |

1. **PRO-CTCAE™ Symptom Term:** Difficulty swallowing

a. In the last 7 days, what was the SEVERITY of your DIFFICLTY SWALLOWING at its WORST?

| Ο None | Ο Mild | Ο Moderate | Ο Severe | Ο Very severe |
| --- | --- | --- | --- | --- |

1. **PRO-CTCAE™ Symptom Term:** Shortness of breath

a. In the last 7 days, what was the SEVERITY of your SHORTNESS OF BREATH at its WORST?

| Ο None | Ο Mild | Ο Moderate | Ο Severe | Ο Very severe |
| --- | --- | --- | --- | --- |

b. In the last 7 days, how much did your SHORTNESS OF BREATH INTERFERE with your usual or daily activities?

| Ο Not at all | Ο A little bit | Ο Somewhat | Ο Quite a bit | Ο Very much |
| --- | --- | --- | --- | --- |

1. **PRO-CTCAE™ Symptom Term:** PAIN OVER SALIVARY GLANDS

a. In the last 7 days, what was the SEVERITY of your PAIN OVER SALIVARY GLANDS at its WORST?

| Ο None | Ο Mild | Ο Moderate | Ο Severe | Ο Very severe |
| --- | --- | --- | --- | --- |

1. **PRO-CTCAE™ Symptom Term:** Dry mouth

a. In the last 7 days, what was the SEVERITY of your DRY MOUTH at its WORST?

| Ο None | Ο Mild | Ο Moderate | Ο Severe | Ο Very severe |
| --- | --- | --- | --- | --- |

1. **PRO-CTCAE™ Symptom Term:** Mouth/throat sores

a. In the last 7 days, what was the SEVERITY of your MOUTH OR THROAT SORES at their WORST?

| Ο None | Ο Mild | Ο Moderate | Ο Severe | Ο Very severe |
| --- | --- | --- | --- | --- |

b. In the last 7 days, how much did MOUTH OR THROAT SORES INTERFERE with your usual or daily activities?

| Ο Not at all | Ο A little bit | Ο Somewhat | Ο Quite a bit | Ο Very much |
| --- | --- | --- | --- | --- |

1. **PRO-CTCAE™ Symptom Term:** ORAL PAIN

a. In the last 7 days, what was the SEVERITY of your ORAL PAIN at its WORST?

| Ο None | Ο Mild | Ο Moderate | Ο Severe | Ο Very severe |
| --- | --- | --- | --- | --- |

1. **PRO-CTCAE™ Symptom Term:** Taste changes

a. In the last 7 days, what was the SEVERITY of your PROBLEMS WITH TASTING FOOD OR DRINK at their WORST?

| Ο None | Ο Mild | Ο Moderate | Ο Severe | Ο Very severe |
| --- | --- | --- | --- | --- |

1. **PRO-CTCAE™ Symptom Term:** Nausea

a. In the last 7 days, how OFTEN did you have NAUSEA?

| Ο Never | Ο Rarely | Ο Occasionally | Ο Frequently | Ο Almost constantly |
| --- | --- | --- | --- | --- |

b. In the last 7 days, what was the SEVERITY of your NAUSEA at its WORST?

| Ο None | Ο Mild | Ο Moderate | Ο Severe | Ο Very severe |
| --- | --- | --- | --- | --- |

1. **PRO-CTCAE™ Symptom Term:** Vomiting

a. In the last 7 days, how OFTEN did you have VOMITING?

| Ο Never | Ο Rarely | Ο Occasionally | Ο Frequently | Ο Almost constantly |
| --- | --- | --- | --- | --- |

b. In the last 7 days, what was the SEVERITY of your VOMITING at its WORST?

| Ο None | Ο Mild | Ο Moderate | Ο Severe | Ο Very severe |
| --- | --- | --- | --- | --- |

1. **PRO-CTCAE™ Symptom Term:** Heartburn

a. In the last 7 days, how OFTEN did you have HEARTBURN?

| Ο Never | Ο Rarely | Ο Occasionally | Ο Frequently | Ο Almost constantly |
| --- | --- | --- | --- | --- |

b. In the last 7 days, what was the SEVERITY of your HEARTBURN at its WORST?

| Ο None | Ο Mild | Ο Moderate | Ο Severe | Ο Very severe |
| --- | --- | --- | --- | --- |

1. **PRO-CTCAE™ Symptom Term:** Abdominal pain

a. In the last 7 days, how OFTEN did you have PAIN IN THE ABDOMEN (BELLY AREA)?

| Ο Never | Ο Rarely | Ο Occasionally | Ο Frequently | Ο Almost constantly |
| --- | --- | --- | --- | --- |

b. In the last 7 days, what was the SEVERITY of your PAIN IN THE ABDOMEN (BELLY AREA) at its WORST?

| Ο None | Ο Mild | Ο Moderate | Ο Severe | Ο Very severe |
| --- | --- | --- | --- | --- |

c. In the last 7 days, how much did PAIN IN THE ABDOMEN (BELLY AREA) INTERFERE with your usual or daily activities?

| Ο Not at all | Ο A little bit | Ο Somewhat | Ο Quite a bit | Ο Very much |
| --- | --- | --- | --- | --- |

1. **PRO-CTCAE™ Symptom Term:** Dry eyes

a. In the last 7 days, what was the SEVERITY of your DRY EYES at its WORST?

| Ο None | Ο Mild | Ο Moderate | Ο Severe | Ο Very severe |
| --- | --- | --- | --- | --- |

1. **PRO-CTCAE™ Symptom Term:** Watery eyes

a. In the last 7 days, what was the SEVERITY of your WATERY EYES (TEARING) at their WORST?

| Ο None | Ο Mild | Ο Moderate | Ο Severe | Ο Very severe |
| --- | --- | --- | --- | --- |

b. In the last 7 days, how much did WATERY EYES (TEARING) INTERFERE with your usual or daily activities?

| Ο Not at all | Ο A little bit | Ο Somewhat | Ο Quite a bit | Ο Very much |
| --- | --- | --- | --- | --- |

1. **PRO-CTCAE™ Symptom Term:** Painful red eyes

a. In the last 7 days, what was the SEVERITY of your PAINFUL RED EYES at its WORST?

| Ο None | Ο Mild | Ο Moderate | Ο Severe | Ο Very severe |
| --- | --- | --- | --- | --- |
